# Clinical and molecular significance of the RNA m^6^A methyltransferase complex in prostate cancer

**DOI:** 10.3389/fgene.2022.1096071

**Published:** 2023-01-12

**Authors:** Jennifer Lothion-Roy, Daisy B. Haigh, Anna E. Harris, Veronika M. Metzler, Mansour Alsaleem, Michael S. Toss, Yousif Kariri, Atara Ntekim, Brian D. Robinson, Francesca Khani, Lorraine J. Gudas, Cinzia Allegrucci, Victoria H. James, Srinivasan Madhusudan, Melissa Mather, Richard D. Emes, Nathan Archer, Rupert G. Fray, Emad Rakha, Jennie N. Jeyapalan, Catrin S. Rutland, Nigel P. Mongan, Corinne L. Woodcock

**Affiliations:** ^1^ Biodiscovery Institute, University of Nottingham, Nottingham, United Kingdom; ^2^ School of Veterinary Medicine and Sciences, University of Nottingham, Sutton Bonington Campus, Loughborough, United Kingdom; ^3^ School of Medicine, University of Nottingham, Nottingham, United Kingdom; ^4^ Department of Applied Medical Science, Applied College, Qassim University, Qassim, Saudi Arabia; ^5^ Department of Clinical Laboratory Science, Faculty of Applied Medical Science, Shaqra University, Shaqra, Saudi Arabia; ^6^ Department of Radiation Oncology, University Hospital Ibadan, University of Ibadan, Ibadan, Nigeria; ^7^ Department of Pathology, Weill Cornell Medicine, New York, NY, United States; ^8^ Department of Pharmacology, Weill Cornell Medicine, New York, NY, United States; ^9^ Faculty of Engineering, University of Nottingham, Nottingham, United Kingdom; ^10^ School of Biosciences, University of Nottingham, Sutton Bonington Campus, Loughborough, United Kingdom

**Keywords:** METTL3, METTL14, WTAP, CBLL1, splicing, transcriptome

## Abstract

N^6^-methyladenosine (m^6^A) is the most abundant internal mRNA modification and is dynamically regulated through distinct protein complexes that methylate, demethylate, and/or interpret the m^6^A modification. These proteins, and the m^6^A modification, are involved in the regulation of gene expression, RNA stability, splicing and translation. Given its role in these crucial processes, m^6^A has been implicated in many diseases, including in cancer development and progression. Prostate cancer (PCa) is the most commonly diagnosed non-cutaneous cancer in men and recent studies support a role for m^6^A in PCa. Despite this, the literature currently lacks an integrated analysis of the expression of key components of the m^6^A RNA methyltransferase complex, both in PCa patients and in well-established cell line models. For this reason, this study used immunohistochemistry and functional studies to investigate the mechanistic and clinical significance of the METTL3, METTL14, WTAP and CBLL1 components of the m^6^A methyltransferase complex in PCa specimens and cell lines. Expression of METTL3 and CBLL1, but not METTL14 and WTAP, was associated with poorer PCa patient outcomes. Expression of METTL3, METTL14, WTAP and CBLL1 was higher in PCa cells compared with non-malignant prostate cells, with the highest expression seen in castrate-sensitive, androgen-responsive PCa cells. Moreover, in PCa cell lines, expression of METTL3 and WTAP was found to be androgen-regulated. To investigate the mechanistic role(s) of the m^6^A methyltransferase complex in PCa cells, short hairpin RNA (shRNA)-mediated knockdown coupled with next generation sequencing was used to determine the transcriptome-wide roles of METTL3, the catalytic subunit of the m^6^A methyltransferase complex. Functional depletion of *METTL3* resulted in upregulation of the androgen receptor (AR), together with 134 AR-regulated genes. *METTL3* knockdown also resulted in altered splicing, and enrichment of cell cycle, DNA repair and metabolic pathways. Collectively, this study identified the functional and clinical significance of four essential m^6^A complex components in PCa patient specimens and cell lines for the first time. Further studies are now warranted to determine the potential therapeutic relevance of METTL3 inhibitors in development to treat leukaemia to benefit patients with PCa.

## Introduction

Prostate cancer (PCa) is the second most commonly diagnosed male cancer type globally and was estimated to be responsible for 375,000 deaths in 2020 (Sung et al., 2021). While there are a number of treatment options for PCa patients, these are often associated with side effects that negatively impact quality of life, and the emergence of treatment resistance is common (Cornford et al., 2017; Mottet et al., 2017). Indeed, there is no curative therapy for metastatic PCa, however androgen deprivation therapies (ADT) delay disease progression. ADT deprives PCa cells of the stimulatory effects of androgen hormones, either by directly reducing circulating androgen levels, or *via* AR-signalling inhibitors (ARSi) that block the ability of the androgen receptor (AR) to bind its ligand, thereby attenuating AR-dependent tumour growth (Attar et al., 2009). Despite initial efficacy, where ∼80% of patients experience tumour regression following ADT, most patients will progress to a castrate-resistant PCa (CRPC) state where ADT is no longer effective (Gravis et al., 2013; Sweeney et al., 2015).

ADT resistance develops *via* several mechanisms, including increased AR expression and through the expression of aberrant AR variants, such as AR-V7, which lack the ligand binding domain (Sharp et al., 2019). Multiple mechanisms contribute to the expression of AR-variants including aberrant splicing (Antonarakis et al., 2016; Sarwar et al., 2016; Schweizer and Plymate, 2016; Armstrong et al., 2019; Tagawa et al., 2019; Gjyrezi et al., 2021), and these AR variants are associated with CRPC progression, metastasis and poorer overall survival (Mostaghel et al., 2011; Hu et al., 2012). The role of aberrant splicing is also increasingly recognised in the development of neuroendocrine PCa (NEPC) (Lee et al., 2018; Labrecque et al., 2021) with ADT-suppression implicated in treatment-emergent neuroendocrine-like PCa (Li et al., 2013; Van Etten et al., 2017; Wang et al., 2017). Outcomes for patients with CRPC or NEPC remain poor (Beltran et al., 2016; Zou et al., 2017; Zhang et al., 2018; Dong et al., 2020). Given the importance of aberrant gene regulation and splicing in PCa initiation, progression and outcome, pharmacological modulation of gene expression is an attractive target. There is therefore an urgent clinical need to develop novel therapeutic approaches to prevent, delay or reverse PCa progression associated with existing ADTs.

The most prevalent internal covalent modification of messenger RNA (mRNA) in eukaryotes is the methylation of the N^6^ position of adenosine on cellular mRNA, also known as N^6^-methyladenosine (m^6^A) (Desrosiers et al., 1974; Jia et al., 2011; Mongan et al., 2019). The addition of m^6^A occurs co-transcriptionally in the nucleus (Horiuchi et al., 2013; Ping et al., 2014) and is mediated by the RNA m^6^A methyltransferase complex (also known as the m^6^A writers) (Su et al., 2022). This complex includes the methyltransferase-like 3 (METTL3) catalytic subunit bound in a stable complex with methyltransferase-like 14 (METTL14) (Liu et al., 2014). Wilms’ tumour-1 associated protein (WTAP) binds to and stabilises the METTL3-METTL14 heterodimer (Ping et al., 2014; Selberg et al., 2019). WTAP was originally discovered in association with the Wilms’ tumour suppressor gene (*WT1*) (Little et al., 2000), where it was found to partner with WT1 and regulate cell proliferation and quiescence (Penalva et al., 2000; Small et al., 2007). WTAP is thought to recruit RNA that has been targeted for m^6^A modification, facilitates binding by the METTL3-METTL14 complex, and is implicated in the regulation of splicing (Liu et al., 2014; Ping et al., 2014; Su et al., 2022). WTAP has been found to recruit a number of adaptor proteins to the complex to facilitate mRNA processing including VIRMA (KIAA1429), the RBM15 and RBM15B RNA binding proteins, and the ZC3H13 zinc finger protein (Liu et al., 2014; Schwartz et al., 2014; Patil et al., 2016; Wen et al., 2018). Further adaptor proteins are associated with this methyltransferase complex of proteins, including Cbl proto-oncogene like 1 (CBLL1), which is believed to be required for full m^6^A methylation activity (Horiuchi et al., 2013; Ruzicka et al., 2017; Wen et al., 2018; Yue et al., 2018; Sun et al., 2019). CBLL1, also known as HAKAI, is a ubiquitin E3 ligase that regulates E-cadherin expression (Fujita et al., 2002). CBLL1 was later found to be involved in m^6^A regulation as part of the METTL3-METTL14-WTAP complex (Horiuchi et al., 2013; Ruzicka et al., 2017).

The m^6^A modification of mRNA is essential for normal development and function across a wide range of species (Fray and Simpson, 2015; Geula et al., 2015; Haussmann et al., 2016; Ruzicka et al., 2017; Dai et al., 2018; Zhang et al., 2022), suggesting evolutionary conservation of mechanisms involving m^6^A. The m^6^A modification plays a vital role in gene expression, translation and splicing, and has been found to be involved in regulating a number of key normal physiological processes such as embryogenesis, circadian rhythms, response to DNA damage, and cell differentiation (Fustin et al., 2013; Wang et al., 2014; Geula et al., 2015; Lin et al., 2017; Xiang et al., 2017). Although the precise contribution of m^6^A to alternative splicing, exon selection and transcript stability has been debated (Ke et al., 2015; Ke et al., 2017; Darnell et al., 2018; Zhao et al., 2018), there is evidence to support a role for m^6^A in regulating context-dependent splicing *in vivo* (Haussmann et al., 2016; Lence et al., 2016). Dysregulation of alternative splicing has been identified in PCa and was associated with disease progression (Jiménez-Vacas et al., 2020; Zhang et al., 2020). Furthermore, dysregulation of the m^6^A modification has been associated with a number of haematological and solid cancers (Wang et al., 2020b) and implicated in the development of therapy resistance through a range of mechanisms (Li B. et al., 2020).

METTL3 has been reported to have both oncogenic and tumour suppressor roles in a number of cancer types. An oncogenic role for METTL3 has been suggested in several cancers, with high levels of METTL3 expression reported in acute myeloid leukaemia (AML), gastric cancer and hepatocellular carcinoma (HCC) (Barbieri et al., 2017; Vu et al., 2017; Chen et al., 2018; Wei et al., 2022). In addition to its m^6^A methyltransferase function, METTL3 has also been shown to promote the translation of oncogenes, such as epidermal growth factor receptor (*EGFR*) in lung cancer, *via* interaction with ribosomes and the translation initiation complex (Lin et al., 2016). In contrast, *METTL3* has demonstrated a tumour suppressive role in other cancer types such as renal cell carcinoma (RCC) and colorectal cancer (Li et al., 2017; Deng et al., 2019). A number of recent studies have revealed that METTL3 is upregulated in PCa, and that this increased expression promotes migration and invasion of PCa cells, tumour growth, and is associated with higher tumour stage and poorer prognosis (Cai et al., 2019; Ma Z. et al., 2020; Yuan et al., 2020). Altered METTL3 expression has also been associated with therapy resistance in PCa (Cotter et al., 2021). Given the implication of METTL3 in the development and progression of numerous cancer types, there has been considerable interest in small molecule METTL3 inhibitors, that have recently demonstrated preclinical proof of concept evidence for the treatment of leukaemia (Yankova et al., 2021). Such METTL3 inhibitors may also have utility in solid tumours, including PCa (Haigh et al., 2022).

Like METTL3, METTL14 has been shown to act both as a tumour suppressor and an oncogene in regulating the development of a number of different tumour types (Guan et al., 2022). It functions as a tumour suppressor gene in many types of cancer, including liver, kidney, bladder, endometrial and glioblastoma, gastric, colorectal and rectal cancer (Yang et al., 2020; Cai et al., 2021; Zhou et al., 2021; Fan et al., 2022). Oncogenic functions for *METTL14* have been suggested in several cancer types, including pancreatic, breast and AML (Weng et al., 2018; Paris et al., 2019; Wang et al., 2020a; Kong et al., 2020; Yi et al., 2020). In PCa, higher METTL14 expression has been correlated with poor prognosis in PCa patients, with knockdown of *METTL14* attenuating tumour proliferation both *in vitro* and *in vivo* (Wang et al., 2022). Furthermore, upregulation of *METTL14* has been demonstrated to enhance the invasion and metastatic potential of PCa cells (Liu et al., 2022).

An oncogenic function has been described for *WTAP*, though it has not yet been fully established whether this is through its association with WT1 or the METTL3-METTL14 m^6^A methyltransferase complex (Zhang et al., 2016; Knuckles et al., 2018). *WTAP* expression is regulated by METTL3, with its oncogenic function in some cancer types found to be related to its m^6^A methyltransferase complex function (Sorci et al., 2018). Recent studies have reported an overexpression of *WTAP* in bladder cancer (Chen and Wang, 2018) and AML (Bansal et al., 2014). However conflicting findings have been reported in breast cancer, where *WTAP* expression has been reported to be both increased and decreased, as well as not significantly changed, across different stages and molecular subtypes (Christgen et al., 2007; Wu et al., 2019). The relevance of *WTAP* in PCa remains poorly understood. Two recent studies of distinct publicly available PCa patient genomic data sets reported no significant difference in *WTAP* expression between normal prostate and tumour tissue (Wu et al., 2021; Liu et al., 2022). Ji and colleagues reported that *WTAP* expression was significantly higher in PCa (Ji et al., 2020) and that lower *WTAP* expression was associated with elevated Gleason score, suggesting complex roles for WTAP in tumour initiation and progression (Ji et al., 2020). Further work is needed to better understand the role of *WTAP* in PCa initiation and progression.

Whilst CBLL1 is known to regulate E-cadherin, a protein with a key role in the development of cancer (Alsaleem et al., 2019), little is known about the role of CBLL1 in carcinogenesis. CBLL1 expression is markedly elevated in gastric, colon and non-small cell lung cancers (NSCLC), suggesting a pro-oncogenic role (Figueroa et al., 2009; Castosa et al., 2018; Hui et al., 2019). However, the relative importance of its E-cadherin and m^6^A methyltransferase complex functions in PCa remains poorly understood.

Given the involvement of m^6^A in the development and progression of numerous cancer types, this study aimed to further the understanding of the expression, function and clinical relevance of the METTL3, METTL14, WTAP and CBLL1 components of the m^6^A methyltransferase complex in PCa specimens and cell lines. The basal and androgen-regulated mRNA and protein expression of METTL3, METTL14, WTAP and CBLL1 in PCa cell lines was also analysed. Finally, the effect of functional depletion of the *METTL3* catalytic subunit on gene expression and splicing in PCa cells was determined. This study provides new insights into the expression and function of METTL3, METTL14, WTAP and CBLL1 in PCa, and their potential relevance as prognostic indicators and therapeutic targets.

## Materials and methods

### Bioinformatic analysis of METTL3, METTL14, WTAP and CBLL1 expression in PCa patient specimens

Expression and genetic alterations of *METTL3*, *METTL14*, *WTAP* and *CBLL1* were analysed in the publicly-available primary adenocarcinoma TCGA Firehose Legacy cohort (*n* = 499) (Liu J. et al., 2018), metastatic adenocarcinoma SU2C/PCF Dream Team cohort (*n* = 444) (Abida et al., 2019), and NEPC Multi-Institute (*n* = 114) datasets (Beltran et al., 2014) using the cBioPortal for Cancer Genomics (Cerami et al., 2012; Gao et al., 2013) (retrieved 24 June 2022).

Analysis of mRNA expression of *METTL3*, *METTL14*, *WTAP* and *CBLL1* in primary prostate adenocarcinoma compared with non-malignant prostate tissue was carried out in the GDC TCGA Prostate Cancer (PRAD) dataset (Liu J. et al., 2018) using the UCSC Xena browser (Goldman et al., 2020) (retrieved 24 June 2022).

### PCa specimens and immunohistochemistry

A prostate tissue microarray (TMA) was constructed from a well-characterised Nottingham University Hospitals (NUH) NHS Trust prostatectomy patient cohort diagnosed with PCa from 2003 to 2007 (Supplementary Table S1). The TMA comprised 104 primary prostate adenocarcinoma and 56 non-malignant tissue specimens, each represented by a .6 mm formalin fixed paraffin embedded (FFPE) core. Full face sections were assessed by a pathologist and representative cores retrieved to construct the TMA.

The study was completed with the approval of the local ethics committee of the University of Nottingham School of Veterinary Medicine and Science (1861161006 and 3483211102) and the NUH NHS Trust Biobank Access Committee (ACP0000184). The General Data Protection Regulation (GDPR) was applied, with the Human Tissue Act and Helsinki Declaration of Human Rights strictly observed.

Immunohistochemical (IHC) staining was performed using the Novolink Max Polymer Detection System (Leica Biosystems, United Kingdom) as previously described (Kariri et al., 2021). The TMA block was cut into 4 µm sections and stained with primary antibodies: anti-METTL3 (ab195352 PUR; Abcam, 1:500), anti-METTL14 (ab220030; Abcam, 1:3,000), anti-WTAP (NBP1-83040; Novus Biologicals, 1:400), anti-CBLL1 (NBP1-83589; Novus Biologicals, 1:250). All primary antibodies were incubated for 1 h at room temperature. The stained slides were subsequently scanned at high resolution with the Nanozoomer (Hamamatsu Photonics, United Kingdom; METTL3, WTAP and CBLL1) or with the Pannoramic 250 Flash III (3D Histech, Hungary; METTL14).

Nuclear and cytoplasmic staining was assessed using the semi-quantitative histo-score (H-score) system [0–300 range: 0 × (% cells with no staining 0) + 1 × (% cells with weak staining intensity 1) + 2 × (% cells with moderate staining intensity 2) + 3 × (% cells with strong staining intensity 3)] (McCarty et al., 1985). To compare protein expression between PCa and non-malignant prostate tissue, expression was only assessed in malignant cells in tumour cores; and in non-malignant glandular epithelial cells in the non-malignant cores. H-scores were validated by a second scorer, who independently assessed a minimum of 10% of all specimens and confirmed concordance of >.75 *via* intraclass correlation. Cytoplasmic staining for METTL3, METTL14, and WTAP was not observed above background levels and were therefore not analysed.

H-scores were categorised into high, medium, and low expression groups for each protein and correlated with clinicopathological parameters. METTL3 nuclear staining was assessed in non-malignant (*n* = 39) and tumour samples (*n* = 92) and divided into H-score groups of low (≤115), medium (116–155) and high (≥156). METTL14 nuclear staining was assessed in non-malignant (*n* = 42) and tumour samples (*n* = 68) and divided into H-score groups of low (≤110), medium (111–149) and high (≥150). WTAP nuclear staining was assessed in non-malignant (*n* = 41) and tumour samples (*n* = 93) and divided into H-score groups of low (≤100), medium (101–129) and high (≥130). CBLL1 nuclear staining was assessed in non-malignant (*n* = 43) and tumour samples (*n* = 99) and divided into H-score groups of low (≤89), medium (90–100) and high (≥101), with CBLL1 cytoplasmic staining grouped as low (≤30), medium (31–70) and high (≥71).

### Cell culture

This study utilised six prostate epithelial cell lines: non-malignant PNT1A; AR-expressing, androgen-dependent LNCaP (representing castrate-sensitive prostatic adenocarcinoma); AR-expressing, androgen-independent LNCaP:C4-2 (representing metastatic castrate-resistant PCa); AR-expressing, androgen-independent 22Rv1 representing castrate-resistant, enzalutamide-resistant PCa, which also expresses AR splice variants implicated in ADT resistance and PCa recurrence (Hörnberg et al., 2011; Antonarakis et al., 2014; Qu et al., 2015; Scher et al., 2016; Steinestel et al., 2019), and androgen-insensitive PC3 and DU145 (representing aggressive castrate-resistant metastatic PCa and do not express AR).

All cell lines were cultured in RPMI-1640 medium supplemented with 10% foetal bovine serum (FBS), 1 mM sodium pyruvate, 100 U/ml penicillin and 100 μg/ml streptomycin and were maintained at 37°C and 5% CO_2_. For androgen treatment in LNCaP, LNCaP:C4-2, and 22Rv1, cells were plated in phenol-red-free RPMI-1640 medium supplemented with 10% charcoal-stripped FBS, 2 mM L-glutamine, 1 mM sodium pyruvate, 100 U/ml penicillin and 100 μg/ml streptomycin. A final concentration of 1 nM R1881 or .1% ethanol (vehicle control) was added in experiments investigating androgen regulation.

HEK-293T cells were used for lentiviral transfections and were cultured in DMEM medium supplemented with 10% FBS, 1 mM sodium pyruvate, 100 U/ml penicillin, 100 μg/ml streptomycin, 1 mM sodium pyruvate, and were maintained at 37°C and 5% CO_2_. All reagents were purchased from Gibco (United States) or Sigma-Aldrich (United States). All experiments were carried out in triplicate.

### shRNA-mediated knockdown of *METTL3*

Inducible lentiviral-mediated knockdown was achieved essentially as described (Orfali et al., 2019). Briefly, two tetracycline (TET)-inducible short hairpin RNAs (shRNAs) identified by the RNAi Consortium (www.broad.mit.edu/genome_bio/trc/rnai.html) and targeting METTL3 expression (clone ID: TRCN0000034716 and TRCN0000034718) were tested. One guide (clone ID: TRCN0000034718), targeting exon 4–5 of *METTL3* achieved optimal METTL3 depletion (Supplementary Figure S10) and was prioritised for detailed characterisation in LNCaP:C4-2 cells. Lentiviral particles were generated in HEK293T cells using either the sh*METTL3* targeting construct (Tet-pLKO-puro sh*METTL3*) or non-gene targeting scramble control (Tet-pLKO-puro-scrambled) with pDR8.91 packaging vector (Nova Lifetech, Singapore), and pVSV-G envelope vector (Addgene, United States) in serum-free Opti-MEM™ (Thermo Fisher Scientific, United States). Following lentiviral-mediated delivery of the shScramble control or sh*METTL3* constructs into LNCaP:C4-2 cells, stable clones were selected by puromycin treatment (1 μg/ml) for up to 21 days. *METTL3* knockdown was induced by treating stable, selected clones with doxycycline (1 μg/ml) for 6 days.

### Analysis of gene expression and western blotting

RNA was extracted with the GenElute Mammalian Total RNA Miniprep Kit with on-column DNase treatment (Sigma-Aldrich, United States) and cDNA synthesised using the qScript cDNA Synthesis Kit (Quantabio, United States). Quantitative real-time polymerase chain reaction (qRT-PCR) was carried out to analyse mRNA expression with either a LightCycler^®^ 480 II instrument (Roche, Switzerland) or a CFX Connect Real-Time PCR Detection System (Bio-Rad Laboratories, United States), using LightCycler^®^ 480 Probes Master (Roche, Switzerland) and hydrolysis probes. The following Taqman probes were used in this study: *METTL3* (Hs00219820_m1), *METTL14* (Hs00383340_m1), *WTAP* (Hs04987070_m1), *CBLL1* (Hs00227265_m1), with β*-actin* (Hs01060665_g1) or *GAPDH* (Hs03929097_g1) used as housekeeping genes (all Thermo Fisher Scientific, United States). mRNA relative expression was calculated using the Pfaffl method (Pfaffl, 2001).

For protein analysis, cells were harvested in final sample buffer (100 mM Tris-HCl pH 6.8, 4% SDS and 20% glycerol). A total of 20 µg of each protein sample was loaded on a 10% SDS-PAGE gel for protein separation. Subsequently, proteins were semi-dry transferred to a polyvinylidene difluoride (PVDF) membrane (.45 µm; Merck, Germany) and blocked using 5% milk. The membrane was then incubated with primary antibody diluted as appropriate overnight at 4°C. The following primary antibodies were used in this study: anti-METTL3 (ab195352 PUR; Abcam, United Kingdom, 1: 10,000), anti-METTL14 (ab220030; Abcam, United Kingdom, 1: 1,000), anti-WTAP (NBP1-83040; Novus Biologicals, United States, 1: 2,500), anti-CBLL1 (NBP1-83589; Novus Biologicals, United States, 1: 1,000) and anti-β*-*actin (sc-130657; Santa Cruz Biotechnology, United States, 1: 50,000 or MA5-15739; Invitrogen, United States, 1: 10,000). Finally, the membrane was incubated in horseradish peroxidase-conjugated secondary antibodies of either goat anti-mouse (ab97023; Abcam, United Kingdom, 1: 10,000–50,000 or Sc-2005; Santa Cruz Biotechnology, United States, 1: 10,000–50,000) or goat anti-rabbit (ab6721; Abcam, United Kingdom, 1: 10,000–50,000 or Sc-2004; Santa Cruz Biotechnology, United States, 1: 10,000–50,000) for 1 h at room temperature. Imaging of the protein signal was carried out using the ChemiDoc™ MP Imaging System (Bio-Rad Laboratories, United States) following a 1-min incubation of the PVDF membrane with Amersham™ ECL™ Prime reagent (GE Healthcare, United States). Full, annotated Western blot images are available in Supplementary Figures S2–S10.

### Differential gene and splicing analysis of *METTL3* depletion in PCa cells

Paired-end RNA sequencing (RNA-seq) of vehicle-treated control and *METTL3*-depleted LNCaP:C4-2 cells was completed using an Illumina NovoSeq instrument (Novogene, United Kingdom). Raw data was analysed using standard approaches. Briefly, the TrimGalore wrapper (https://github.com/FelixKrueger/TrimGalore) for FastQC and Cutadapt (Martin, 2011) was used to remove contaminating adapter sequences and reads with phred < 30 were discarded. Retained fastq reads were aligned to the Ensembl annotated human reference genome (GRCh38.83) using STAR (Dobin et al., 2013), and gene level expression quantified by FeatureCounts (Liao et al., 2014). A minimum of 24 million reads were obtained for each sample (mean = 31,307,381 ± 1,202,115 reads). The mean % of uniquely mapped reads for STAR alignments was 94.79% (±.1073%). Statistically significant differential expression (fold change ≤ ±1.5 and adjusted *p*-value < .05) was determined using DESeq2 (Love et al., 2014). The VolcaNoseR ShinyApp (https://huygens.science.uva.nl/VolcaNoseR/) was used to prepare a volcano plot of differentially expressed genes. Hierarchical clustering analysis of significantly differentially expressed AR-regulated genes (Sharma et al., 2013) was completed using Cluster 3.0 (de Hoon et al., 2004).

The replicate multivariate analysis of transcript splicing (rMATS version 3.2.5) tool was used to identify differential canonical and *de novo* splicing. Briefly, reads were trimmed to a uniform length (145 bp) using the rMATS trimFastq python script (Shen et al., 2014) and the resultant reads aligned to the Ensembl reference genome (GRCh38.83) using the rMATS implementation of STAR to align reads. Differential splicing was considered biologically and statistically significant based on percent spliced in (dPSI ≥ 5%) and false discovery rate (FDR) < .05. Pathway analysis was completed using the Web-Based Gene Set AnaLysis Toolkit (WebGestalt) (Liao et al., 2019) to identify significantly enriched Kyoto Encyclopaedia of Genes and Genomes (KEGG) pathways (Liao et al., 2019). Genes that were both significantly differentially expressed and alternatively spliced were compared using Venny 2.1.0 (csic.es). Detailed description of RNAseq outputs are available in Supplementary Tables S3–S9. Data is available from NCBI-GEO at the following accession: GSE210130.

### Assessment of phenotypic effects of *METTL3* depletion

The effect of *METTL3* knockdown on LNCaP:C4-2 proliferation was examined. The sh*METTL3* and shSCR cells were treated with 1 μg/ml doxycycline (Sigma-Aldrich) selection for 6 days and the cell counts completed using the Coulter Counter system. Changes in cellular invasion following *METTL3* knockdown were assessed using a Matrigel (Corning, United States) invasion assay. Matrigel (Corning, United States) diluted in coating buffer was used to coat cell culture inserts (Corning, United States) which were then incubated overnight at 37°C. Cells were seeded in FBS-free, phenol-red-free, RPMI-1640 medium on to the Matrigel coated membrane in the upper chamber of the cell culture inserts. Phenol-red-free RPMI-1640 medium supplemented with 10% FBS was added into the lower well chamber beneath the insert and the cells were incubated at 37°C for 24 h. Invaded cells were fixed with alcohol and stained with .4% crystal violet. Cells were imaged with an inverted microscope (Leica, Germany). The number of cells were then manually assessed and relative invasion calculated compared to control cell lines.

### Statistical analysis

Protein expression *via* western blot was quantified using Image Studio Lite (version 5.2), with statistical analysis of mRNA and protein expression carried out using GraphPad PRISM 9, with t-tests performed to compare two means. All experiments were presented as the mean plus standard error of the mean of two independent experiments. *p*-values < .05 were considered statistically significant.

Analysis of IHC staining of PCa patient TMAs and associated clinicopathological parameters was performed using IBM^®^ SPSS^®^ Statistics (version 28). Statistical *p*-values were determined by χ^2^ test (asymptotic significance, 2-sided) using both IBM^®^ SPSS^®^ Statistics and the VassarStats Statistical Computation Website (http://vassarstats.net/; ©Richard Lowry). *p*-values < .05 were considered statistically significant with a confidence interval of 95%.

This study followed the reporting recommendation for tumour marker prognostic studies (REMARK) criteria (Sauerbrei et al., 2018).

## Results

### METTL3, METTL14, WTAP and CBLL1 expression and genetic alterations in prostate cancer patients

**FIGURE 1:**
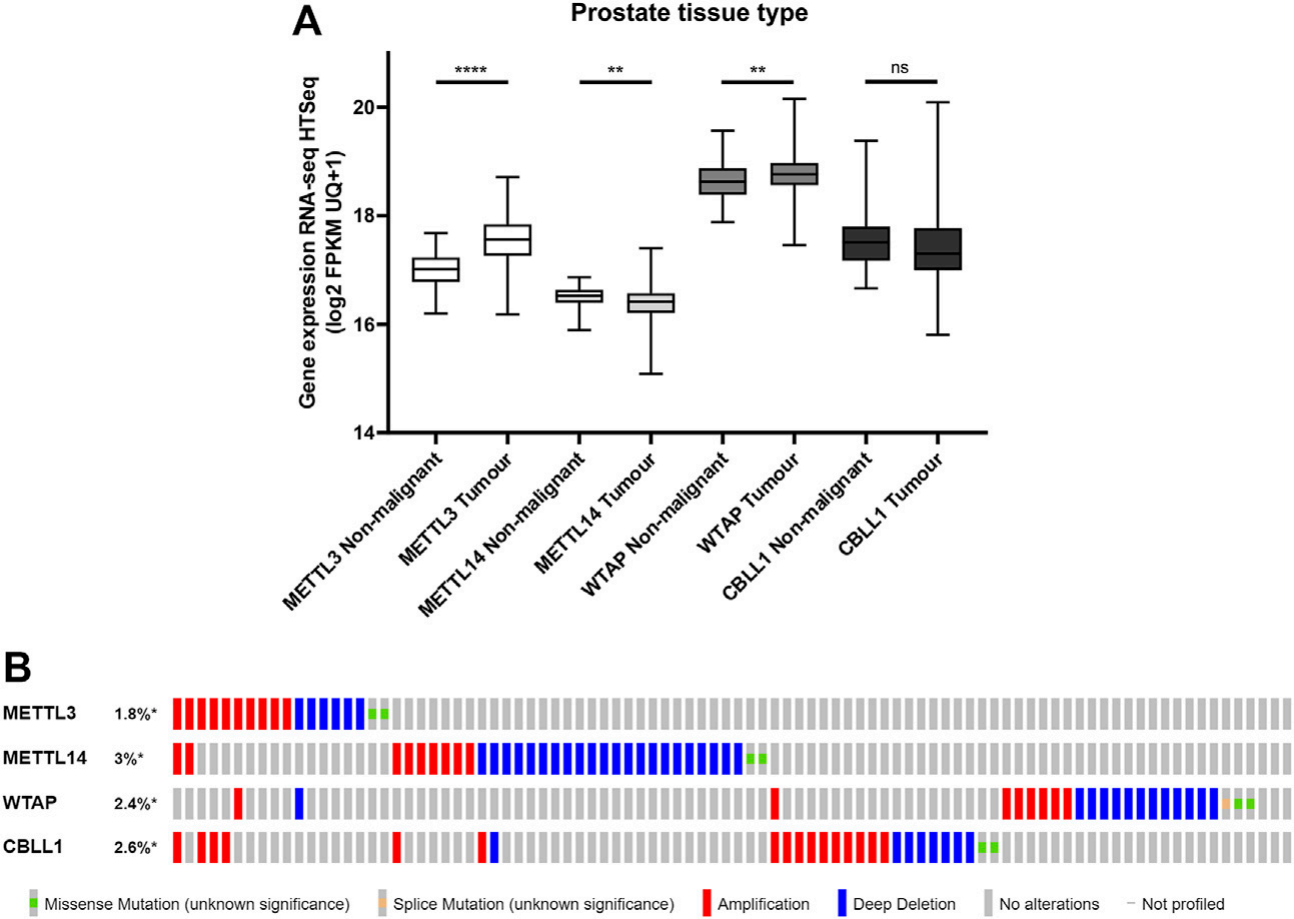
*METTL3, METTL14, WTAP* and *CBLL1* expression in non-malignant and PCa tissue and genetic alteration analysis. Data from the GDC TCGA Prostate Cancer (PRAD) dataset were downloaded from USCS Xena [**(A)**; *n* = 623]. *METTL3, METTL14, WTAP* and *CBLL1* expression in non-malignant prostate tissue was compared with primary prostate adenocarcinoma specimens. The cBioPortal for Cancer Genomics was utilised to assess the incidence of amplification, deep deletion and mutation of *METTL3, METTL14, WTAP* and *CBLL1* in patients with adenocarcinoma, metastatic adenocarcinoma and neuroendocrine PCa [**(B)**; *n* = 1,059]. ns, not significant; **p ≤ .005, ****p ≤ .0001 by unpaired *t*-test.

To evaluate the clinical relevance of proteins from the m^6^A methyltransferase complex in PCa, the mRNA expression of *METTL3*, *METTL14*, *WTAP* and *CBLL1* in publicly available PCa patient datasets was assessed (Figure 1). It was found that *METTL3* (*p* < .0001) and *WTAP* (*p* < .01) expression were significantly higher in primary prostate adenocarcinoma patient samples compared with non-malignant prostate tissue, whereas *METTL14* expression was significantly lower (*p* < .01) in tumour compared with non-malignant tissue (Figure 1A). No significant difference in expression of *CBLL1* was identified in tumour when compared with non-malignant tissue.

A comparative analysis of PCa patients in the primary prostate adenocarcinoma, metastatic prostate adenocarcinoma, and NEPC datasets was undertaken to assess of the frequency of genetic alterations affecting these genes (Figure 1B; Supplementary Figure S1). Expression of *METTL3* was altered in 1.8% of PCa patients, *METTL14* in 3% of patients, *WTAP* in 2.4% of patients and *CBLL1* in 2.6% of patients. Gene amplification was the most common alteration of *METTL3* and *CBLL1* in 10/18 and 16/26 samples, respectively. Deep deletion was the most prevalent genetic alteration in both *METTL14* and *WTAP*, present in 22/33 and 13/24 samples, respectively. It is notable that alteration in expression of *METTL3*, *METTL14*, *WTAP* and *CBLL1* appeared to be mutually exclusive, with 88% of patients having altered expression of only one of these genes.

### METTL3, METTL14, WTAP and CBLL1 expression in prostate cancer patient tissue samples

**FIGURE 2:**
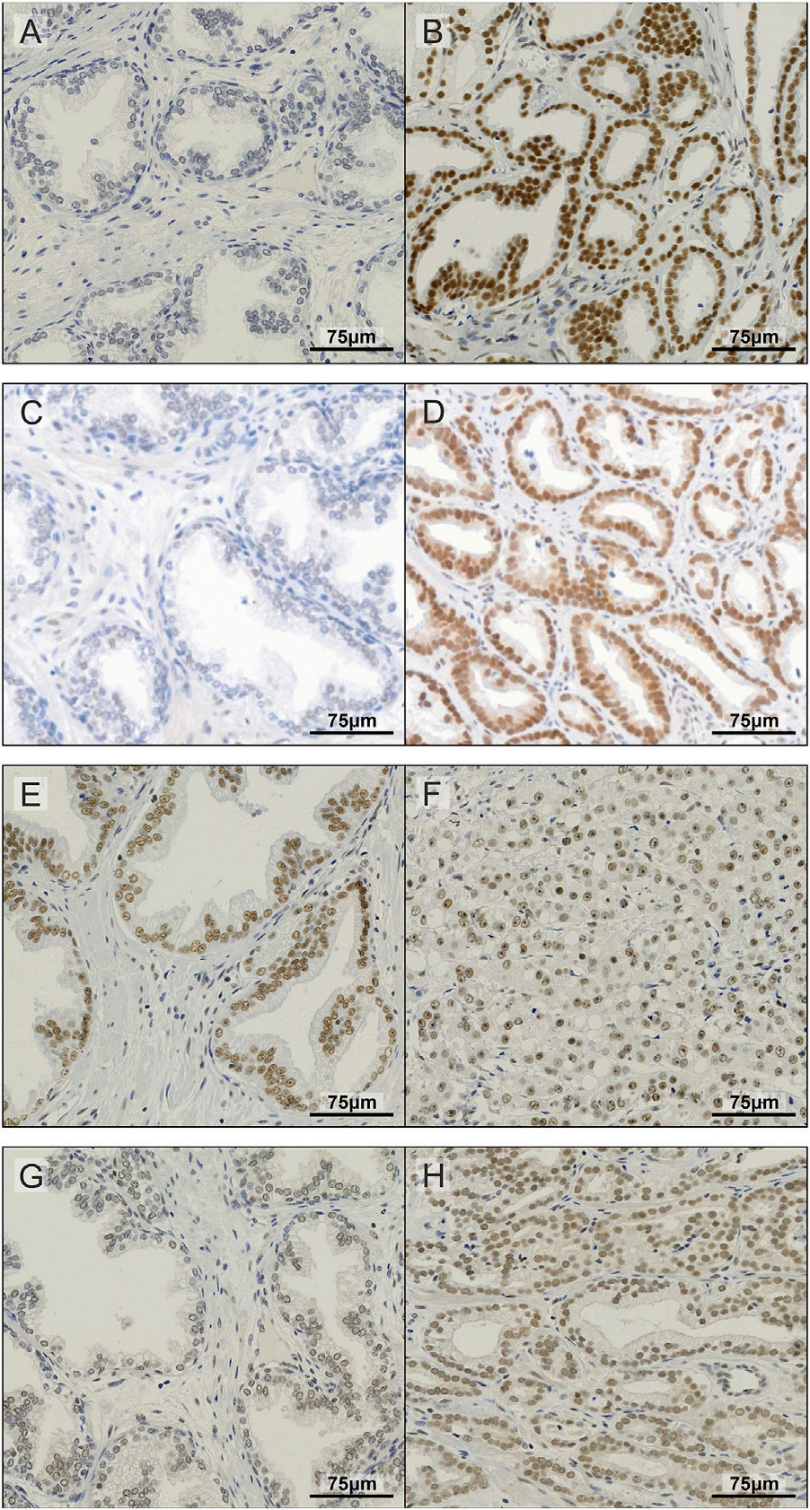
Representative images of IHC staining in prostate TMA. Examples of non-malignant **(A)** and prostate tumour tissue **(B)** stained for METTL3; non-malignant **(C)** and prostate tumour tissue **(D)** stained for METTL14; non-malignant **(E)** and prostate tumour tissue **(F)** stained for WTAP; and non-malignant **(G)** and prostate tumour tissue **(H)** stained for CBLL1.

**FIGURE 3:**
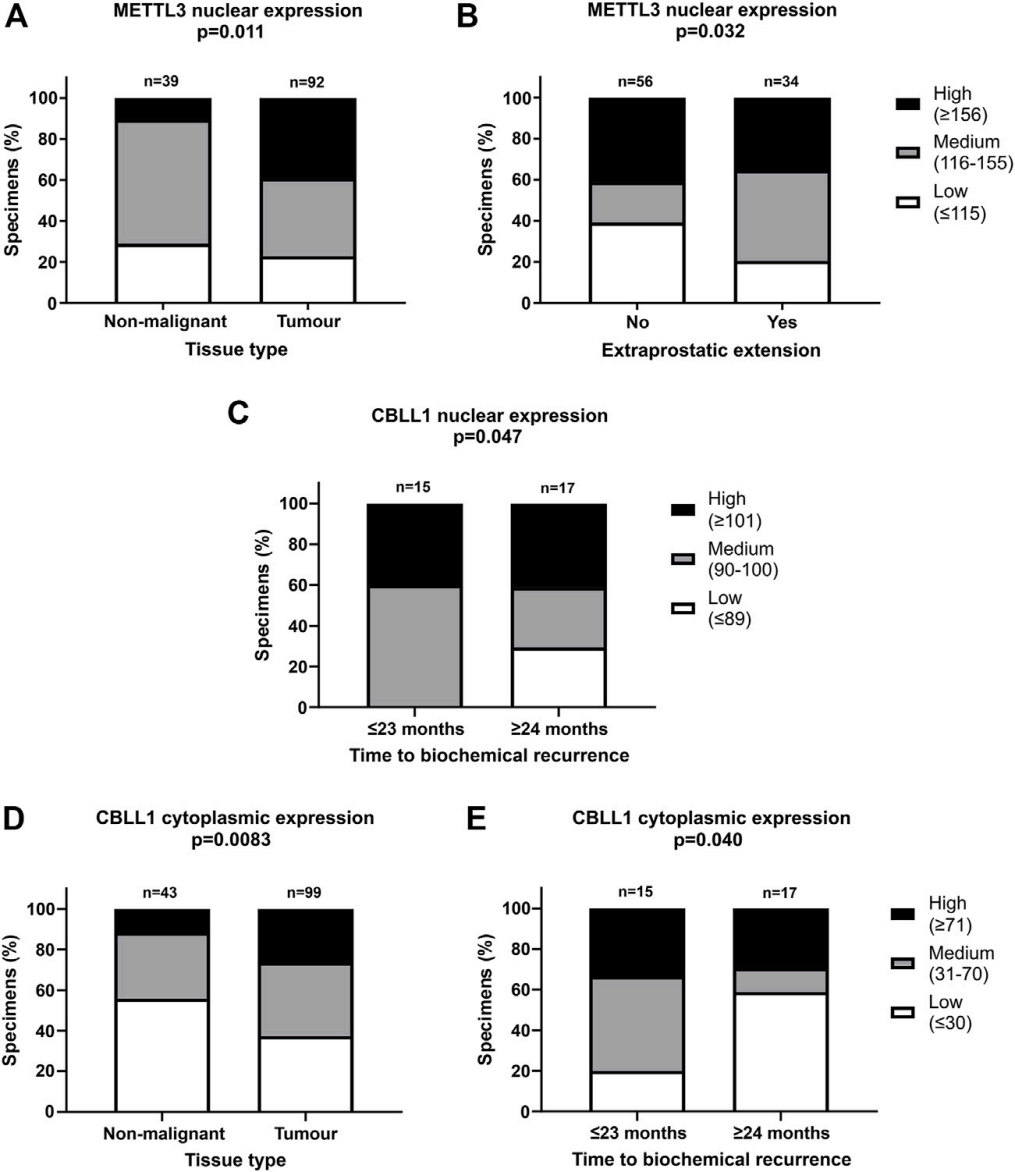
Significant correlations of clinicopathological parameters with METTL3 and CBLL1 expression in prostate cancer TMA. IHC staining of 159 patient samples was assessed by H-score, divided into high, medium, and low expression groups and correlated with prostate tissue type **(A,D)**, extraprostatic extension **(B)** and biochemical recurrence of PCa **(C,E)**. In METTL3 nuclear, low: ≤115, medium: 116–155, high: ≥156; in CBLL1 nuclear, low: ≤89, medium: 90–100, high: ≥101; in CBLL1 cytoplasmic, low: ≤30, medium: 31–70, high: ≥71. Analysed by χ^2^ test.

To further investigate the expression of METTL3, METTL14, WTAP and CBLL1 in PCa patients, IHC was carried out on a prostate TMA comprising non-malignant and tumour specimens (Figure 2) and subsequently correlated with clinicopathological parameters (Figure 3; Supplementary Table S2).

METTL3, METTL14, WTAP and CBLL1 were expressed at the protein level in both non-malignant prostate (Figures 2A, C, E, G) and tumour tissue (Figures 2B, D, F, H). Analysis of protein expression in non-malignant and tumour specimens identified that METTL3 nuclear expression was higher in tumour specimens compared with non-malignant prostate tissue (*p* = .011) (Figure 3A). Similarly, cytoplasmic CBLL1 expression was higher in PCa when compared with non-malignant tissue (*p* = .0083) (Figure 3D). METTL3 nuclear expression was also found to be higher in those patients with extraprostatic tumour extension (*p* = .032), than those without (Figure 3B). It was also found that CBLL1 expression was significantly higher both in the nucleus (*p* = .047) (Figure 3C) and cytoplasm (*p* = .040) (Figure 3E) in patients with PCa recurrence within 2 years, compared with those with later recurrence.

Nuclear CBLL1 expression was higher in patients with a higher Gleason score at diagnosis (*p* = .031) (Supplementary Table S2) yet, interestingly, lower cytoplasmic CBLL1 expression was observed in PCa patients with concurrent high grade prostatic interepithelial neoplasia (PIN) (*p* = .037) (Supplementary Table S2). METTL14 and WTAP expression was not significantly associated with any of the clinicopathological parameters assessed (Supplementary Table S2).

### METTL3, METTL14, WTAP and CBLL1 expression in prostate cell lines

**FIGURE 4:**
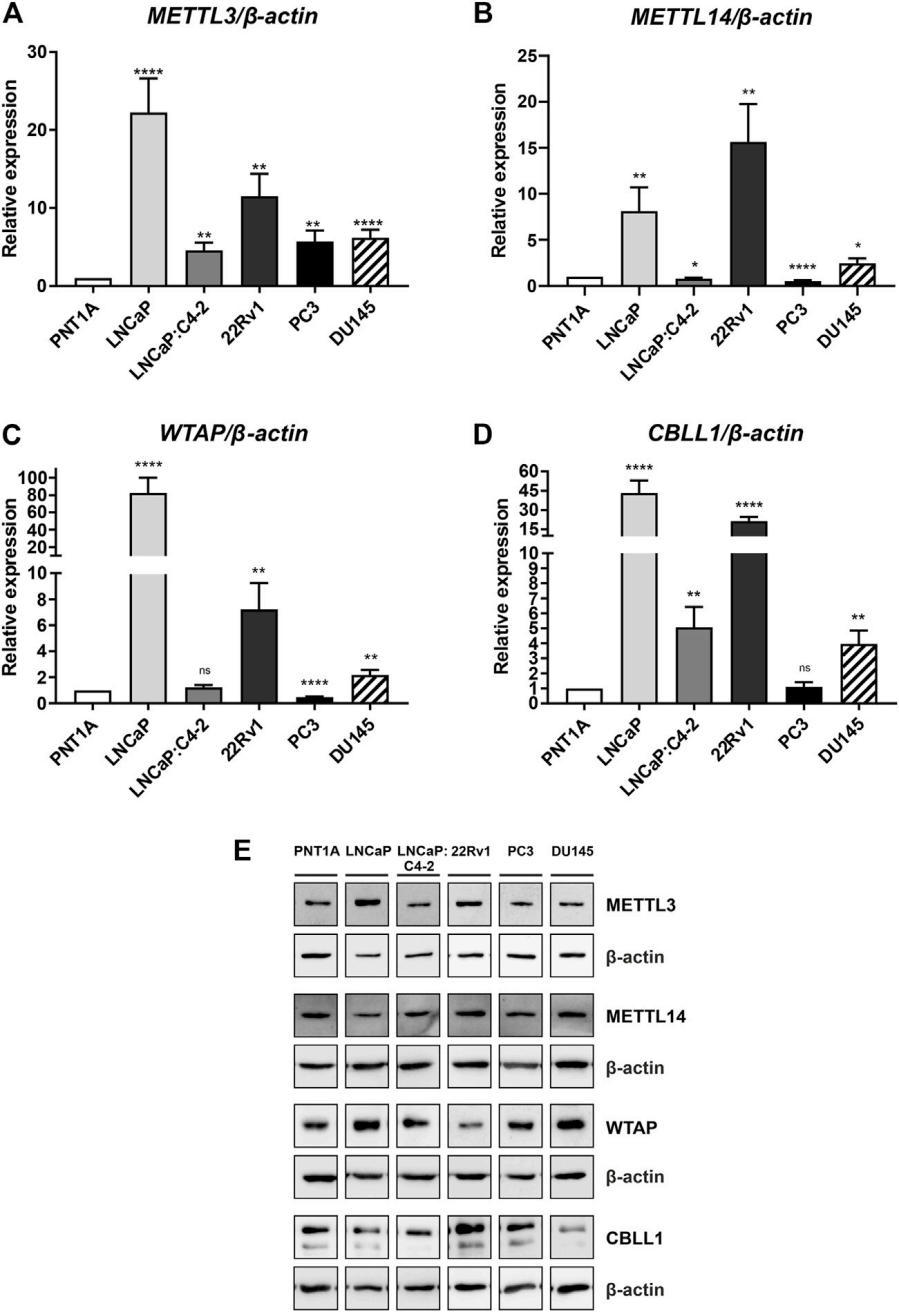
METTL3, METTL14, WTAP and CBLL1 expression in non-malignant and prostate cancer cell lines; mRNA expression analysed by qRT-PCR relative to *β-actin* **(A–D)** and protein expression analysed using western blot **(E)**. METTL3 protein expression as previously reported in Haigh et al. (2022). *p ≤ .05, **p ≤ .005, ***p ≤ .001, ****p ≤ .0001 by unpaired *t*-test.

Basal expression of *METTL3*, *METTL14*, *WTAP* and *CBLL1* was quantified in prostate cell lines at the mRNA and protein level (Figure 4). All four components of the m^6^A methylation complex investigated were expressed at both the mRNA and protein level in all cell lines. *METTL3* expression was significantly higher in all PCa cell lines relative to the non-malignant PNT1A (Figure 4A), as we reported previously (Haigh et al., 2022). Similarly, *CBLL1* expression was found to be significantly higher in all PCa cell lines expressing AR (Figure 4D), with *METTL14* and *WTAP* expression higher in LNCaP, 22Rv1 and DU145 when compared with PNT1A (Figures 4B, C, respectively). The highest levels of mRNA expression of all four genes were seen in the castrate-sensitive LNCaP and castrate-resistant 22Rv1 cell lines (*p* ≤ .01). METTL3 [as previously reported in Haigh et al. (2022)], METTL14, WTAP and CBLL1 protein was expressed in all prostate cell lines examined (Figure 4E). Uncropped western blots are presented (Supplementary Figures S2–S10).

### Androgen regulation of METTL3, METTL14, WTAP and CBLL1

**FIGURE 5:**
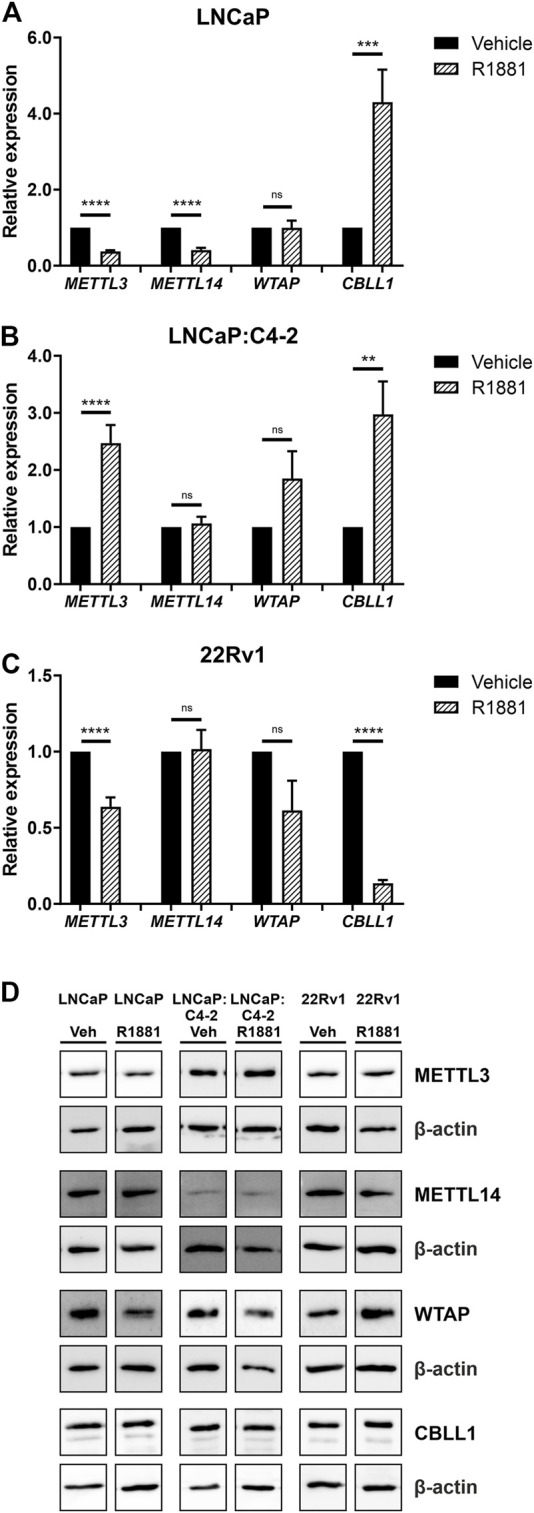
*METTL3*, *METTL14*, *WTAP* and *CBLL1* expression in PCa cell lines LNCaP (*n* = 9) **(A)**, LNCaP:C4-2 (*n* = 9) **(B)**, 22Rv1 (*n* = 6) **(C)** with vehicle or R1881 (1 nM) treatment for 72 h; mRNA expression analysed by qRT-PCR relative to *β-actin* and protein expression analysed using western blot **(D)**. ns, not significant; **p ≤ .005, ***p ≤ .001, ****p ≤ .0001 by unpaired *t*-test.

As expression of all four genes was highest in the androgen-responsive PCa cell lines, the effect of androgen (R1881) on the expression of *METTL3*, *METTL14*, *WTAP* and *CBLL1* mRNA and protein was next examined (Figure 5). *METTL3* expression was significantly downregulated by androgen treatment in LNCaP and 22Rv1 (Figures 5A, C), but upregulated in LNCaP:C4-2 cells (*p* < .0001) (Figure 5B) (Haigh et al., 2022). Similarly, *METTL14* expression was downregulated by androgen treatment in LNCaP (*p* < .0001) (Figure 5A). However, *CBLL1* expression was upregulated in LNCaP (*p* < .001) (Figure 5A) and LNCaP:C4-2 (*p* < .01) (Figure 5B) in response to androgen but downregulated in 22Rv1 cells (*p* < .0001) (Figure 5C). No significant change in *WTAP* expression was identified in response to androgen treatment in any cell line (Figures 5A–C).

At the protein level, METTL3 protein expression was significantly downregulated in response to androgen treatment in LNCaP, whilst WTAP protein expression was significantly upregulated in LNCaP:C4-2 (Figure 5D). No significant changes in protein expression of METTL14 or CBLL1 were seen in response to androgen treatment in any cell line (Figure 5D).

### Differential gene expression, alternative splicing and alterations to cellular phenotype following functional depletion of *METTL3*

**FIGURE 6:**
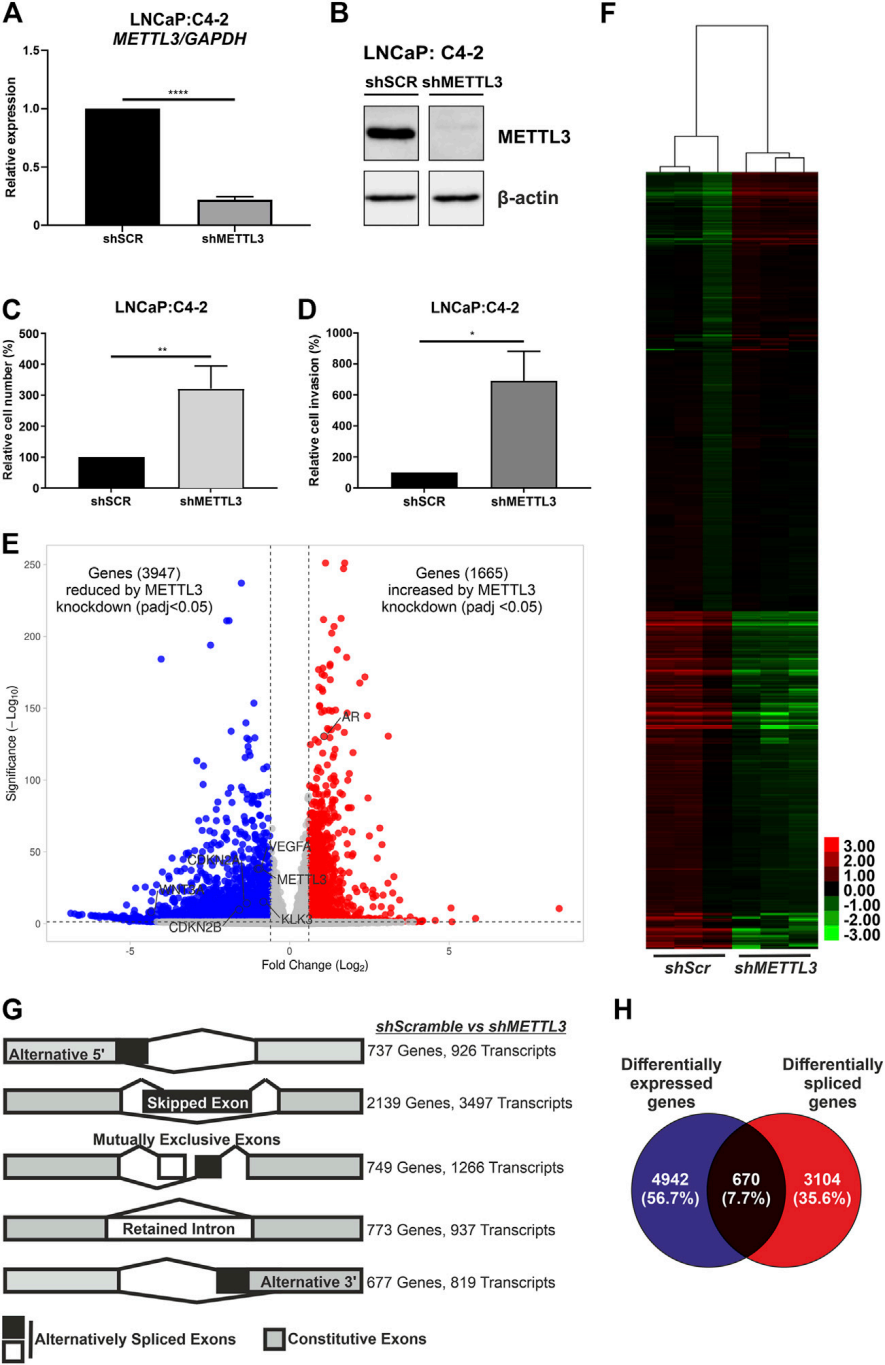
Inducible shRNA-mediated knockdown of *METTL3* in LNCaP:C4-2 cells. Confirmation of *METTL3* knockdown in LNCaP:C4-2 cells at the RNA **(A)** and protein **(B)** level. Cellular proliferation **(C)** and invasion **(D)** in sh*METTL3*-treated LNCaP:C4-2 cells compared with shSCR scrambled controls. Distribution of differentially expressed genes between shSCR and sh*METTL3*-treated LNCaP:C4-2 cells **(E)**. Heatmap of differentially expressed AR-regulated genes **(F)**. Differential alternative splicing determined by rMATS analysis. Transcripts refers to total number of transcripts observed, whereby multiple different transcripts can be observed for a single gene, and the overall number of genes differentially spliced. Alternative splicing events considered: alternative 5′ splice site (A5SS), skipped exon (SE), mutually exclusive exons (MXE) and retained intron (RI) and alternative 3′ splice site (A3SS) **(G)**. Differentially expressed genes compared with differentially spliced genes **(H)**. ns, not significant; *p ≤ .05, **p ≤ .005, ****p ≤ .0001 by unpaired *t*-test. DEG significant if fold change ≤ ±1.5 and adjusted *p*-value < .05 and splicing event significant if dPSI ≥ 5% and FDR < .05.

The mRNA (Figure 6A) and protein (Figure 6B) expression of METTL3 was reduced in doxycycline-treated cells transduced with shRNA targeting *METTL3* compared with shScramble control cells. RNA-seq was used to determine the effect of *METTL3* depletion on gene expression and splicing. This analysis identified that 5612 genes were significantly differentially expressed between shScramble and *shMETTL3*-treated LNCaP:C4-2 cells (Figure 6E; Supplementary Tables S3–S9). Functional depletion of *METTL3* resulted in the upregulation of 1665 genes including, most notably in the context of PCa, *AR* (Figure 6E)*. VEGFA, KLK3, WNT3A, CDKN2B* and *CDKN2A* were among the 3,947 genes downregulated following *METTL3* knockdown (Figure 6E). Indeed expression and splicing of *VEGFA* regulated by *METTL3* was evidenced by RNA-seq (Supplementary Figure S11). Additionally, 134 *AR*-regulated genes were significantly differentially expressed at the transcript level when *METTL3* was depleted (Figure 6F; Supplementary Table S6). *METTL3* depletion was also found to significantly alter splicing (Figure 6G; Supplementary Table S5). Significant alterations were observed in all splicing events investigated; 926 alternative 5′ splice site (A5SS) events, 3497 skipped exon (SE) events, 1,266 mutually exclusive exon (MXE) events, 937 retained intron (RI) events and 819 alternative 3′ splice site (A3SS) events (Figure 6G). SE was the most frequently observed alternative splicing event following *METTL3* depletion with 3497 occurrences affecting a total of 2,139 genes. Of the 5,612 differentially expressed genes, 670 (7.7%) were also alternatively spliced (Figure 6H).

Pathway analysis of differentially expressed genes identified key metabolic and cell cycle-associated pathways that were significantly enriched following *METTL3* depletion (Supplementary Table S4). Similarly, metabolic pathways were significantly enriched in the genes observed to have A5SS, SE, MXE and RI splicing events (Supplementary Table S5). The spliceosome pathway was also enriched in A5SS, SE and RI event gene sets. No pathways were significantly enriched in the gene set of A3SS events.

Knockdown of *METTL3* using shRNA in LNCaP:C4-2 cells resulted in significantly increased cellular proliferation compared with shSCR controls (*p* < .005) (Figure 6C). Likewise, significantly increased invasion of cells was observed following *METTL3* knockdown (*p* < .05) (Figure 6D).

## Discussion

Numerous recent studies have implicated m^6^A and its writer proteins in many cancer types, including in PCa (Bansal et al., 2014; Vu et al., 2017; Liu M. et al., 2018; Paris et al., 2019; Wang et al., 2020a; Zhou et al., 2020). However, there is currently a lack of detailed clinical and mechanistic understanding of the expression of the distinct methyltransferase components in PCa specimens (Cotter et al., 2021; Haigh et al., 2022). Therefore, this study aimed to further elucidate the expression, function and clinical relevance of METTL3, METTL14, WTAP and CBLL1 in PCa patient specimens and cell lines.

Analysis of publicly available PCa patient datasets identified that *METTL3* and *WTAP* expression was higher in primary prostate adenocarcinoma than in non-malignant prostate tissue, whereas conversely, *METTL14* expression was found to be significantly lower. No significant difference was observed in *CBLL1* expression between PCa and non-malignant tissue.

A comparative analysis of genetic alterations in *METTL3*, *METTL14*, *WTAP* and *CBLL1* in primary prostate adenocarcinoma and metastatic PCa patients was undertaken. No genetic alterations were identified in the NEPC patient cohort. It is notable that alteration in expression of these four genes appeared to be largely mutually exclusive of one another, with 88% of patients having altered expression of only one gene.

Gene amplification was the most common alteration observed in *METTL3.* It is interesting that whilst the majority of patients showed altered expression of only a single gene, 60% of patients that had amplified *METTL3* expression had concurrent amplification of at least one of the other three methyltransferase complex genes assessed. Moreover, all patients with multiple gene amplifications originated from the metastatic PCa cohort. These findings are consistent with an oncogenic function of *METTL3*, with higher expression associated with PCa, a more advanced disease stage and poorer prognosis (Cai et al., 2019; Ji et al., 2020; Ma Z. et al., 2020; Yuan et al., 2020; Haigh et al., 2022).

In contrast, the most frequently detected genetic alteration of *METTL14* in PCa patients was deep deletion. These findings suggest that *METTL14* may have a tumour suppressor function, as previously reported for other cancer types, including gastrointestinal and urological tract malignancies, and glioblastoma (Tao et al., 2020; Yang et al., 2020; Cai et al., 2021; Zhou et al., 2021; Fan et al., 2022). However, recent studies have suggested that *METTL14* can have a pro-tumourigenic role in PCa (Ji et al., 2020; Liu et al., 2022; Wang et al., 2022). *METTL14* has also been implicated as an oncogene in breast cancer, another endocrine-driven cancer (Yi et al., 2020), as well as in pancreatic cancer and AML (Weng et al., 2018; Paris et al., 2019; Kong et al., 2020; Wang et al., 2020b). Further mechanistic work is now warranted to dissect the functional contribution and clinical relevance of METTL14 in PCa.

Interestingly, deep deletion was also the most frequent alteration observed in 54% (13/24) of cases harbouring a *WTAP* alteration, affecting both prostate adenocarcinoma (7/9) and metastatic PCa patients (6/15). *WTAP* amplification was only observed in metastatic PCa patients (8/15). Consistent with this, previous studies have identified that *WTAP* can function as both an oncogene and tumour suppressor dependent on cancer type (Bansal et al., 2014; Chen and Wang, 2018; Wu et al., 2019) and it may be that WTAP functions differently in varying stages of PCa. The limited studies of WTAP suggest a complex, context-dependent role in PCa (Ji et al., 2020; Wu et al., 2021; Liu et al., 2022). There is also evidence that WTAP and METTL3 form a mutual regulatory network, with *METTL3* knockdown reported to affect WTAP expression in AML cells (Sorci et al., 2018).

Amplification of *CBLL1* was the most common genetic alteration found in PCa patients. Of those patients with amplified *CBLL1*, a majority (88%) were from the metastatic PCa cohort, suggesting that higher expression of *CBLL1* may be associated with more advanced PCa. Whilst little is currently known about the role of *CBLL1* in PCa, these findings may suggest an oncogenic function. This is supported by reports of increased expression of *CBLL1* in other cancer types (Figueroa et al., 2009; Castosa et al., 2018; Hui et al., 2019) and that *CBLL1* knockdown results in arrested cell cycle and reduced cellular proliferation (Horiuchi et al., 2013).

Expression and sub-cellular localisation of *METTL3*, *METTL14*, *WTAP* and *CBLL1* were confirmed using IHC in both non-malignant and primary prostate adenocarcinoma specimens. Consistent with other studies, only nuclear expression of METTL14 and WTAP was observed (Ping et al., 2014; Wei et al., 2022). Similarly, only nuclear METTL3 expression was observed in the prostate specimens, despite cytoplasmic METTL3 expression having been described elsewhere (Lin et al., 2016; Wei et al., 2022). Given that the focus of this study is the nuclear RNA methyltransferase function of the METTL3 complex, (Horiuchi et al., 2013; Ping et al., 2014; Selberg et al., 2019), only nuclear expression of METTL3, METTL14 and WTAP was analysed. Consistent with its reported dual role in both nuclear m^6^A methylation (Ruzicka et al., 2017) and cytoplasmic regulation of E-cadherin (Fujita et al., 2002), expression of CBLL1 was detected and analysed in both nuclear and cytoplasmic compartments.

In agreement with a number of other studies in distinct PCa cohorts (Ji et al., 2020; Li B. et al., 2020; Yuan et al., 2020; Haigh et al., 2022), METTL3 expression was significantly higher in PCa compared with non-malignant prostate tissue in the current study. Elevated METTL3 expression was also associated with extraprostatic extension, and by inference associated with poorer patient outcomes (Ball et al., 2015). This is consistent with previous PCa studies that have found METTL3 to be associated with disease progression (Ma X.X. et al., 2020; Yuan et al., 2020).

Nuclear CBLL1 expression was higher in patients with a higher Gleason score at diagnosis, a poor prognostic indicator (Gleason, 1966; Gleason and Mellinger, 1974; Gordetsky and Epstein, 2016). Furthermore, higher nuclear and cytoplasmic CBLL1 expression was also associated with earlier PCa recurrence following surgery. Taken together, these findings suggest that the oncogenic functions of CBLL1 identified in other cancer types (Castosa et al., 2018; Liu M. et al., 2018; Liu Z. et al., 2018; Hui et al., 2019) are also relevant to PCa.

No significant association between METTL14 expression was found with any of the clinicopathological parameters assessed in the Nottingham cohort of non-malignant (*n* = 56) and PCa (*n* = 104) specimens. This contrasts with a previous report from a limited number of non-malignant (*n* = 11) and PCa (*n* = 49) specimens (Wang et al., 2022). Likewise, WTAP expression was not significantly associated with any clinicopathological parameters in the current study, as previously reported (Wu et al., 2021). Collectively our results from patient specimens support a predominant clinical and mechanistic role for METTL3 in PCa.

To determine the translational relevance of PCa cell lines in the study of m^6^A, the expression of *METTL3*, *METTL14*, *WTAP* and *CBLL1* mRNA and protein expression was assessed. The mRNA expression of all four genes was higher in PCa cell lines compared with non-malignant prostate cells, with the highest expression of *METTL3*, *METTL14*, *WTAP* and *CBLL1* observed in the androgen-dependent LNCaP and in castrate-resistant 22Rv1 cells which also express AR variants. In general, expression of *METTL3*, *METTL14*, *WTAP* and *CBLL1* was higher in AR-expressing PCa cells compared with AR-negative PC3 and DU145 cells. Similar findings were also seen at the protein level. This may suggest potential functional interaction between androgen signalling and elevated METTL3 in PCa carcinogenesis, but that METTL3 and, by inference, m^6^A may play a less prominent role in castrate-resistant PCa. Consistent with this, elevated *METTL3* expression has been associated with more advanced tumour stage and a poorer prognosis (Yuan et al., 2020), whereas reduced METTL3 expression has been linked with advanced metastatic PCa (Cotter et al., 2021). Therefore, it is possible that METTL3 may play distinct roles in hormone-dependent and castrate-resistant PCa contexts.

Consistent with previous studies, METTL3 protein expression was higher in both PCa patient specimens and PCa cell lines compared with non-malignant prostate tissue and cells (Cai et al., 2019; Barros-Silva et al., 2020; Li E. et al., 2020; Yuan et al., 2020; Haigh et al., 2022). This supports an oncogenic role for METTL3 in PCa as reported in other cancer types (Barbieri et al., 2017; Vu et al., 2017; Chen et al., 2018).

Whilst some previous studies have indicated that METTL14 can have a tumour suppressor role in certain cancer contexts and types (Yang et al., 2020; Cai et al., 2021; Fan et al., 2022), the current study identified elevated *METTL14* expression in PCa cell lines compared with the non-malignant control. These findings are consistent with recent studies that have suggested a pro-tumourigenic role for METTL14 in PCa (Cai et al., 2019; Barros-Silva et al., 2020; Li E. et al., 2020). Indeed, it has been reported that ectopic over-expression of *METTL14* in PC3 cells increased cell proliferation, migration and invasion, whereas knockdown of *METTL14* reversed these phenotypes (Tao et al., 2020; Yang et al., 2020; Cai et al., 2021; Zhou et al., 2021; Fan et al., 2022).

To our knowledge the expression of CBLL1 and WTAP in PCa cell lines has not been reported to date. Elevated CBLL1 and WTAP expression in PCa compared with non-malignant prostate cells is consistent with results observed in other cancer types where higher CBLL1 expression has been reported in HCC and NSCLC (Liu M. et al., 2018; Liu Z. et al., 2018), with elevated WTAP expression found in bladder cancer and AML (Bansal et al., 2014; Chen and Wang, 2018).

As expression of the four components of the m^6^A methyltransferase complex studied here was highest in the androgen-responsive, AR-expressing PCa cell lines, the effect of androgen treatment on METTL3, METTL14, CBLL1 and WTAP expression was examined. Expression of both *METTL3* and *METTL14* was downregulated by androgen, whereas androgen increased *CBLL1* expression in LNCaP cells. Expression of *METTL3* and *CBLL1* were downregulated by androgen in castrate-resistant 22Rv1 cells. However, androgen increased *METTL3* and *CBLL1* expression in the castrate-resistant LNCaP:C4-2. At the protein level, METTL3 expression followed the mRNA expression in LNCaP cells and was significantly downregulated in response to androgen. WTAP protein expression was found to increase following androgen treatment in LNCaP:C4-2 cells, however no significant change in expression was seen at the mRNA level, suggesting post-transcriptional regulation of expression.

These findings highlight a potential role for *METTL3*, *METTL14* and *CBLL1* in gene regulation and splicing in PCa. Their varied expression following androgen treatment highlights the complex regulation of *METTL3*, *METTL14* and *CBLL1* by the AR in hormone-dependent and hormone-independent PCa cell lines. Furthermore, the functional and mechanistic consequences of aberrant basal and androgen-regulated expression of METTL3, METTL14, CBLL1 and WTAP in PCa remain unknown. It is possible that aberrant expression of individual methyltransferase complex components may alter the stoichiometry, composition and function of the m^6^A methyltransferase complex.

To advance understanding of METTL3 and m^6^A in CRPC, RNA sequencing was used to determine the consequences of shRNA-mediated depletion of *METTL3*. Analysis of this sequencing data confirmed a role for METTL3 in gene expression and splicing in CRPC cells. This analysis identified significantly enriched pathways involved in the cell cycle, metabolism and DNA repair. Key pro-oncogenic and cell cycle mediators including *MYC*, *TERT*, *KDM1A*, *CDK1* and *CDK2* were found to be negatively regulated by *METTL3*. *METTL3* knockdown affected genes associated with fatty acid, arginine and proline metabolism, DNA repair and the cell cycle. rRNA m^6^A methylation was also recently implicated in regulation of fatty acid metabolism (Peng et al., 2022). While rRNA m^6^A methylation is mediated by a distinct METTL5-containing methylation complex, the current study found that functional depletion of *METTL3* also disrupted expression of genes involved in lipid metabolism (Supplementary Table S4) suggesting a wider role for m^6^A in metabolic regulation. Our study also found that *METTL3* knockdown increased expression of *PARP1*, *PARP4*, DNA polymerase D1 (*POLD1*) and apurinic/apyrimidinic endodeoxyribonuclease 2 (*APEX2*), *XRCC2* and other key components of DNA repair pathways. There is now considerable interest in the use of PARP inhibitors (PARPi) in PCa. RNA-m^6^A has previously been implicated in resistance to PARPi in ovarian cancer cells (Fukumoto et al., 2019). Furthermore, m^6^A is believed to play a role in the recruitment of DNA polymerase K to sites of DNA damage (Xiang et al., 2017). Thus, METTL3 expression may be useful in determining which patients may benefit from PARPi therapies. Further research is now required to better understand the functional interactions of METTL3, m^6^A and PARPi in PCa.

Most notably in the context of PCa, the expression of *AR* was higher in LNCaP:C4-2 cells where *METTL3* had been depleted. Interestingly, the *AR* transcript was found to harbour m^6^A in LNCaP cells (Cotter et al., 2021). Consistent with this, expression of the AR-target gene *NKX3.1* was also increased following *METTL3* depletion. However, expression of the prostate specific antigen gene (*PSA/KLK3*) was reduced following *METTL3* depletion. This indicates that METTL3 has direct effects on AR expression, and secondary and complex effects on the expression of AR-target genes. Future studies are required to further investigate the effect of *METTL3* depletion on global androgen signalling, however the data obtained to date suggests that METTL3 plays a role in limiting AR expression and function. While METTL3 expression is significantly higher in tumour compared with non-malignant prostate tissue, *METTL3* is deleted in a subset of patients. In such patients, the absence of METTL3 may promote AR signalling and carcinogenesis. AR plays essential roles in PCa carcinogenesis and progression and is therapeutically targeted by ARSi such as enzalutamide. The AR continues to orchestrate pro-oncogenic signalling in CRPC (Sharma et al., 2013), therefore identifying METTL3 as a regulator of AR signalling is mechanistically and clinically significant. Further research is now warranted to explore the contribution of METTL3 to ARSi response.

*METTL3* knockdown also affected expression of key epigenetic regulators such as *KDM1A* which, like AR, is negatively regulated by METTL3. KDM1A promotes PCa initiation and progression and is involved in regulating AR expression and function (Cai et al., 2011; Kashyap et al., 2013; Cai et al., 2014). Furthermore, *METTL3* was found to regulate FTO, an α-ketoglutarate-dependent dioxygenase that functions as an RNA demethylase, responsible for demethylating RNA- m^6^A (Wang P. et al., 2016; Wang X. et al., 2016; Tang et al., 2018; Wei et al., 2018), and the cap adjacent m^6^Am modification (Wei et al., 2018; Mauer et al., 2019). However, *WTAP* expression was not significantly altered by functional depletion of *METTL3*, as has been reported in other cancer cell types (Sorci et al., 2018). Our data suggests a complex mutual regulatory mechanism whereby m^6^A RNA methylation and demethylation, coupled with histone lysine methylation may cooperate in the regulation of AR expression.

These findings identified *METTL3*-regulated genes in castrate-resistant LNCaP:C4-2 cells. Cotter and colleagues recently reported a role for METTL3 in parental, castrate-sensitive LNCaP PCa cells (Cotter et al., 2021). Of the seven genes reported to be significantly regulated by *METTL3* depletion in LNCaP (Cotter et al., 2021), three genes were also similarly regulated by *METTL3* depletion in LNCaP:C4-2. Of the 5,612 genes identified to be significantly altered by *METTL3* knockdown in LNCaP:C4-2, 841 (8%) genes harboured m^6^A in the LNCaP miCLIP dataset reported by Cotter et al. (2021) (Supplementary Table S9), including *VEGFA* (Supplementary Figure S11; Supplementary Tables S5, S9). These findings are consistent with a more prominent role for METTL3 in castrate-resistant PCa cells.

Given the role of RNA m^6^A methylation in splicing (Haussmann et al., 2016; Lence et al., 2016), the effect of *METTL3* depletion on global splicing was examined in LNCaP:C4-2 cells. Knockdown of *METTL3* most commonly affected exon inclusion (2,139 genes), followed by retained introns (773 genes), mutually exclusive exons (749 genes), alternative 5′ (737 genes) and alternative 3′ (677 genes). This is consistent with a role for METTL3 and, by inference, m^6^A in exon suppression (Avgan et al., 2019). Crucially, this splicing role is independent of differential expression as only a minority of significant splicing events (7.7%) occurred in significantly differentially expressed genes. Accordingly, depletion of *METTL3* resulted in altered splicing of distinct gene networks involved in splicing. Interestingly, *METTL3* knockdown also affected 5′ alternative splicing of DNA repair genes reinforcing a functional interaction between METTL3, m^6^A and DNA repair in CRPC cells.

The current study found that lentiviral-mediated shRNA depletion of *METTL3* promoted PCa cell proliferation and invasion in LNCaP:C4-2. A recently reported small molecule METTL3 pharmaco-inhibitor (STM2457) (Yankova et al., 2021) was also found to promote PCa cell invasion (Haigh et al., 2022). However, a number of studies have found that cellular proliferation and invasion are reduced following shRNA knockdown of *METTL3* in LNCaP, PC3 and DU145 PCa cells (Cai et al., 2019; Li E. et al., 2020; Chen et al., 2021). This may indicate distinct roles for METTL3 in LNCaP, PC3 and DU145 cells compared with LNCaP:C4-2.

In conclusion, this study reports the expression of four essential components of the m^6^A methyltransferase complex in patient specimens and cell lines for the first time. We have identified clinicopathological associations of these components that were thus far unknown. Functional depletion of *METTL3* supports a role for METTL3 and m^6^A in gene regulation, splicing and metabolic regulation. Moreover, our findings suggest that m^6^A RNA methylation and demethylation may cooperate with histone lysine methylation to regulate AR expression and DNA repair mechanisms. Further work is now warranted to understand the complex mechanisms underpinning these novel findings and to determine whether pharmacological inhibition of METTL3 by drugs in phase I clinical trials for advanced solid tumours (clinicaltrials.gov: NCT05584111) may be relevant to subsets of PCa patients.

## Data Availability

The data presented in the study are deposited in the NCBI GEO repository, accession number GSE210130.
