# Supplementary figures and images for: Clinical and molecular significance of the RNA m6A methyltransferase complex in prostate cancer
Source: Front Genet. 2023 Jan 12;13:1096071. doi: 10.3389/fgene.2022.1096071 (PMC9887525; doi:10.3389/fgene.2022.1096071)

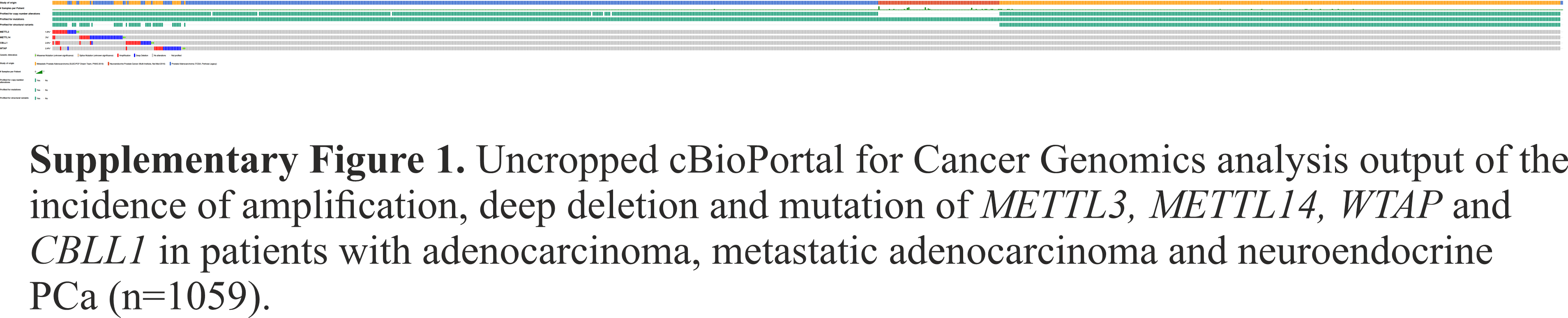

Supplement: Supplementary file 1 [file DataSheet1.zip › Supplementary figures/Supplementary_figure_1.tif]

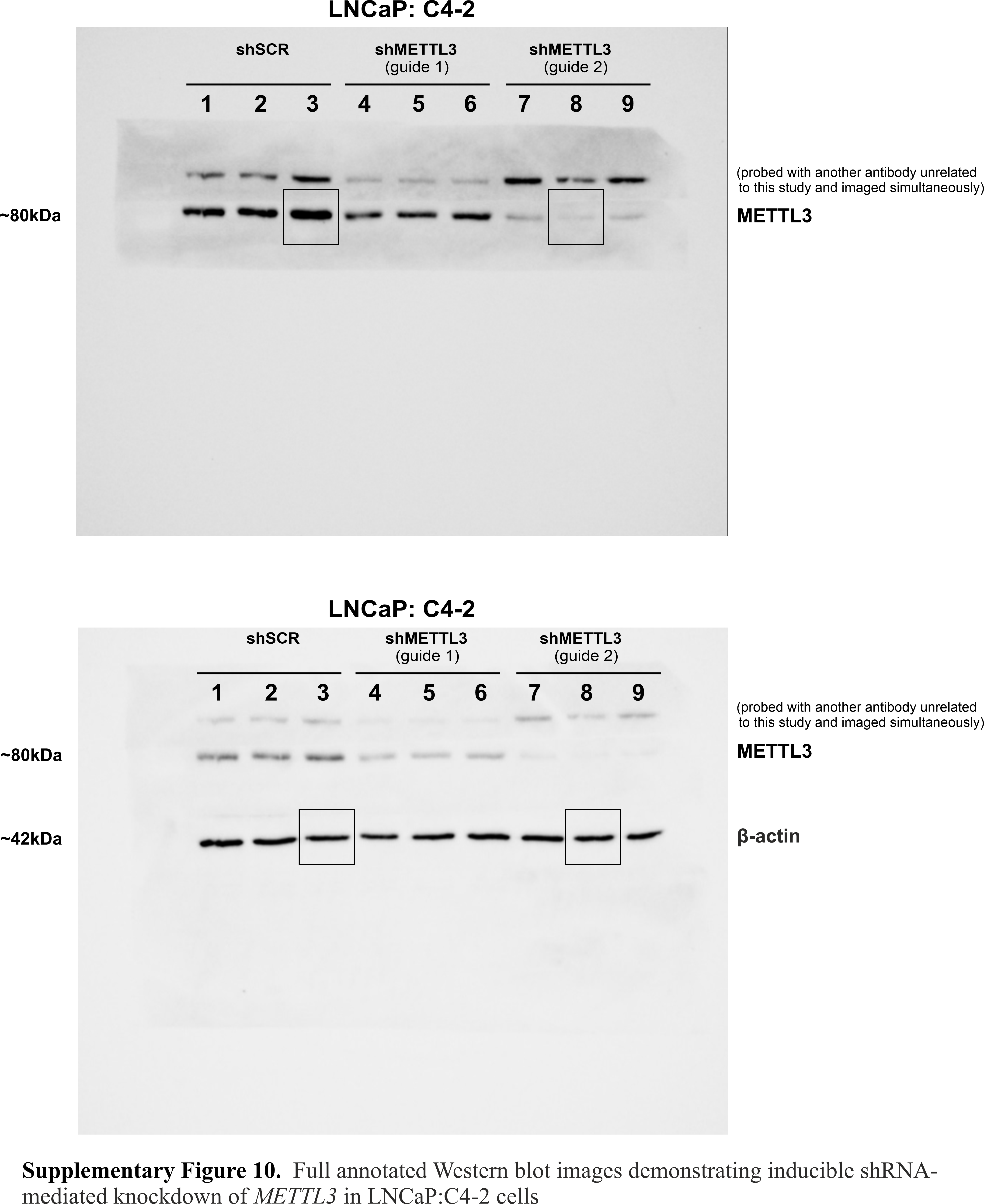

Supplement: Supplementary file 1 [file DataSheet1.zip › Supplementary figures/Supplementary_figure_10.tif]

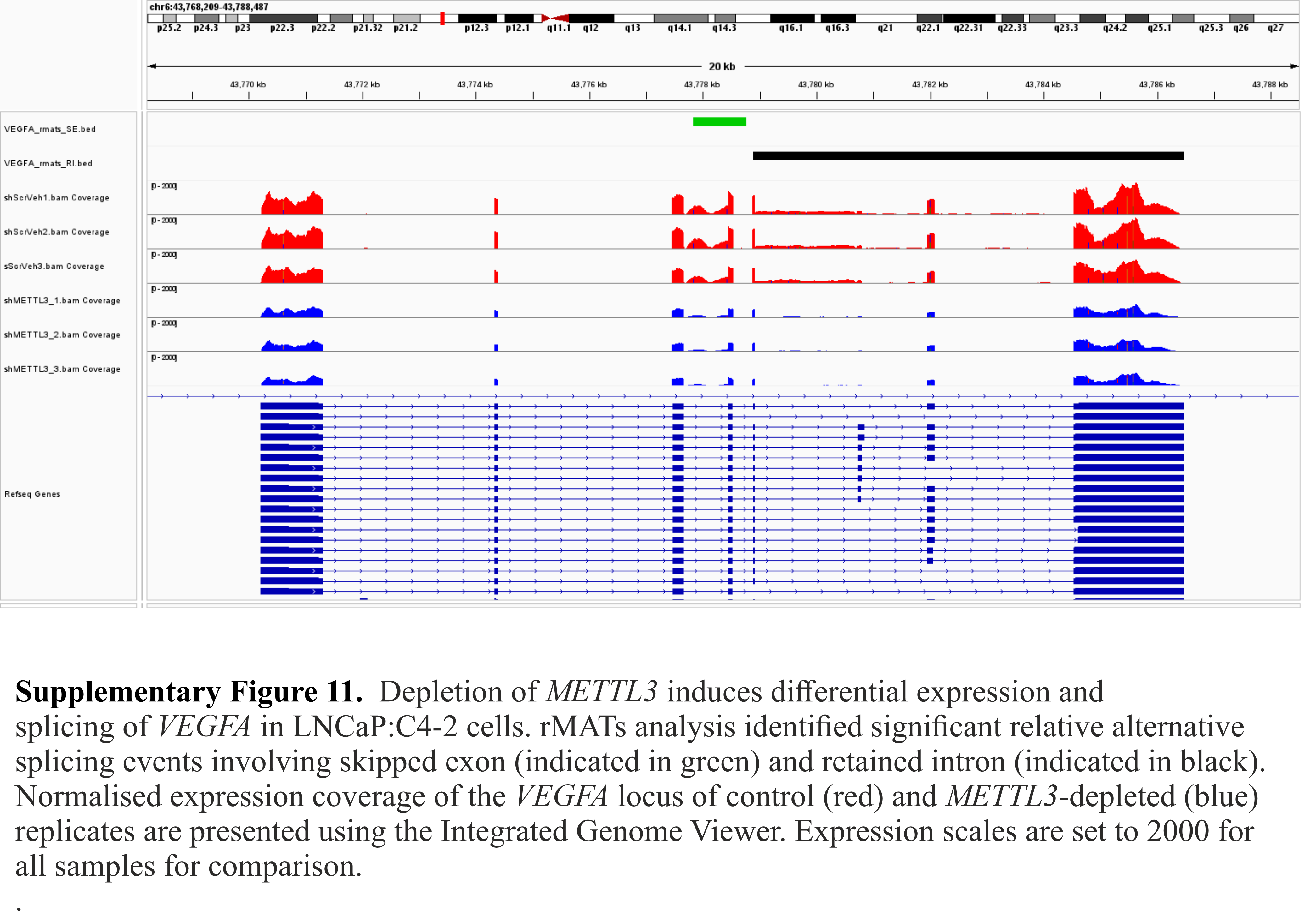

Supplement: Supplementary file 1 [file DataSheet1.zip › Supplementary figures/Supplementary_figure_11.tif]

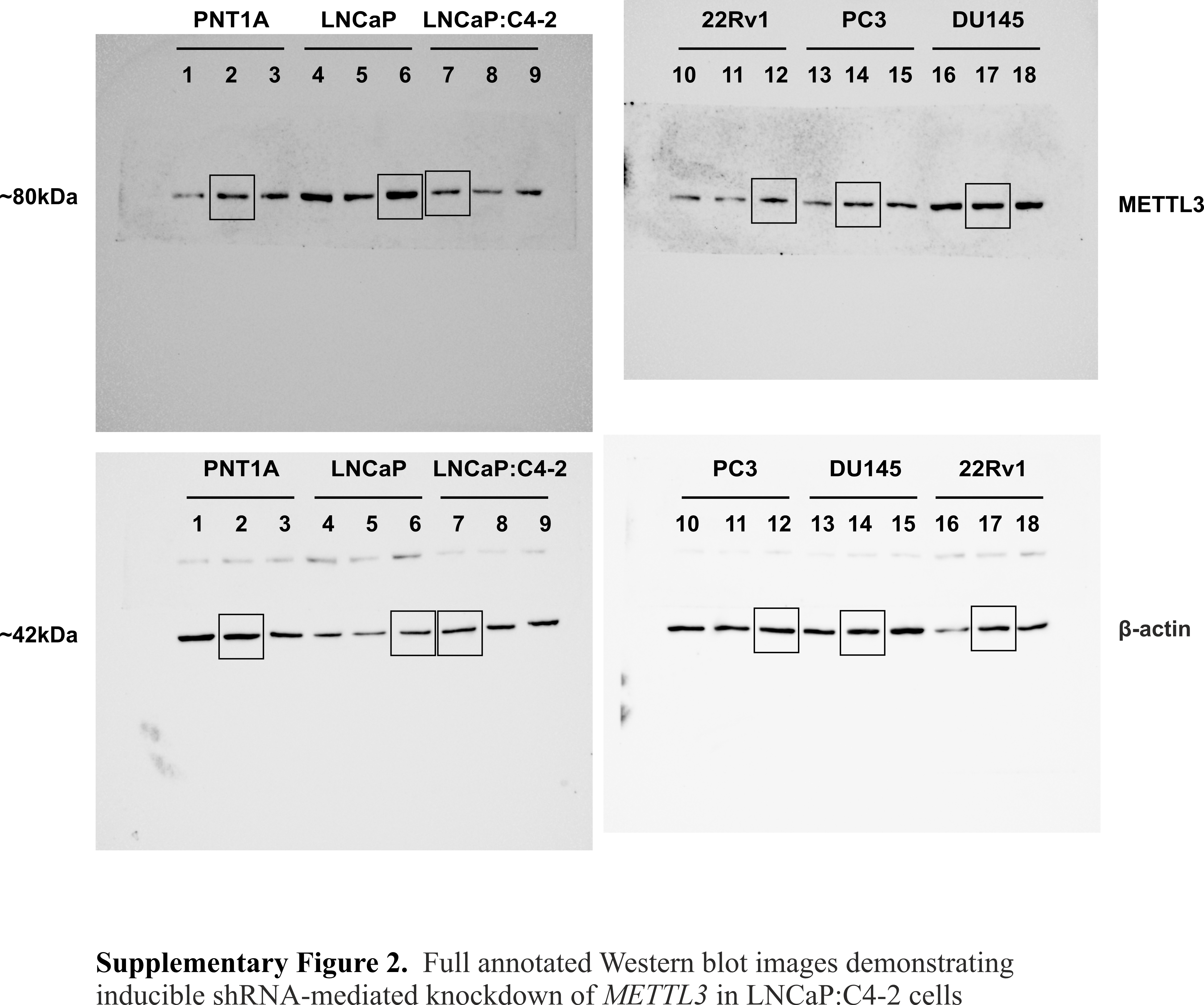

Supplement: Supplementary file 1 [file DataSheet1.zip › Supplementary figures/Supplementary_figure_2.tif]

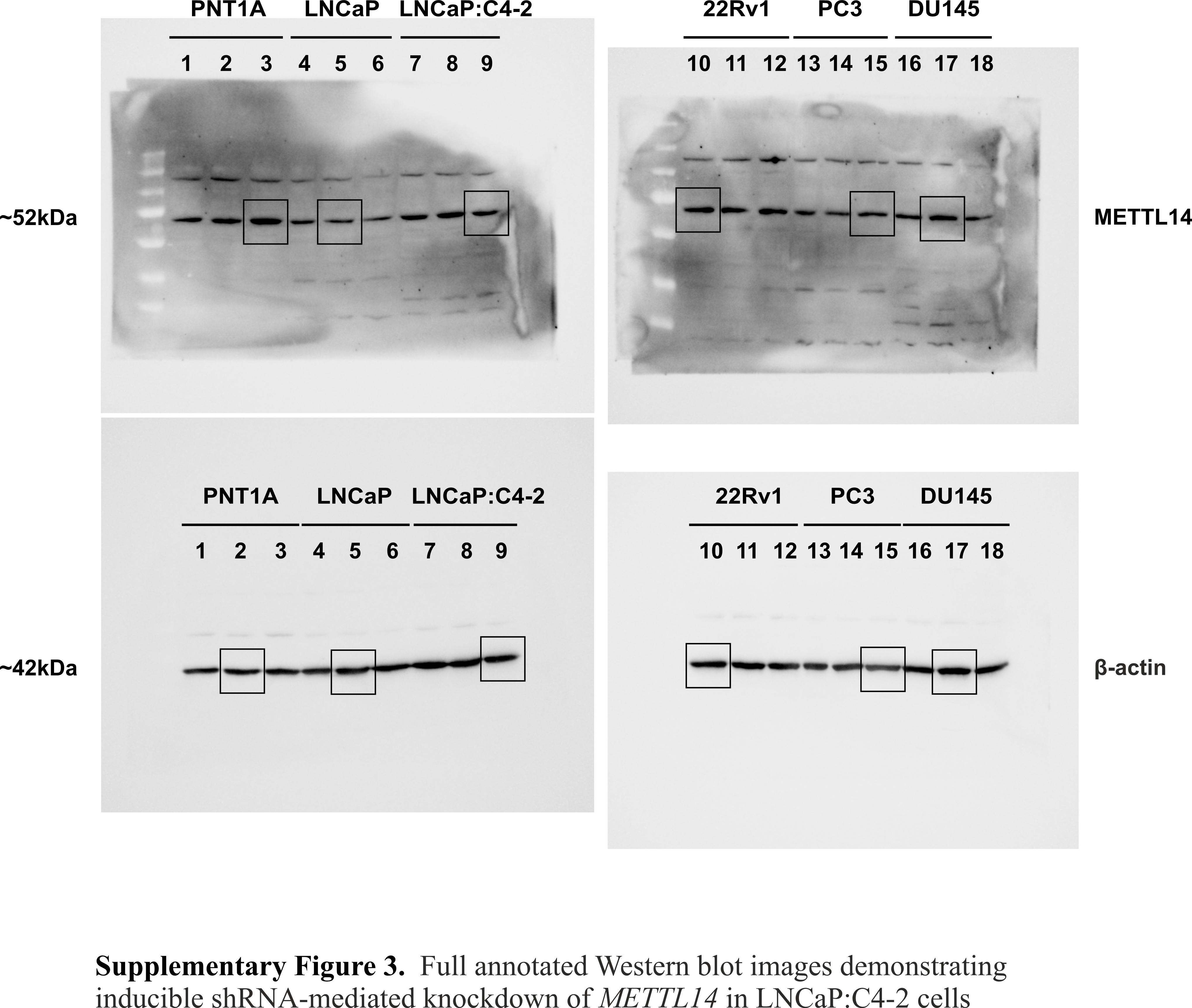

Supplement: Supplementary file 1 [file DataSheet1.zip › Supplementary figures/Supplementary_figure_3.tif]

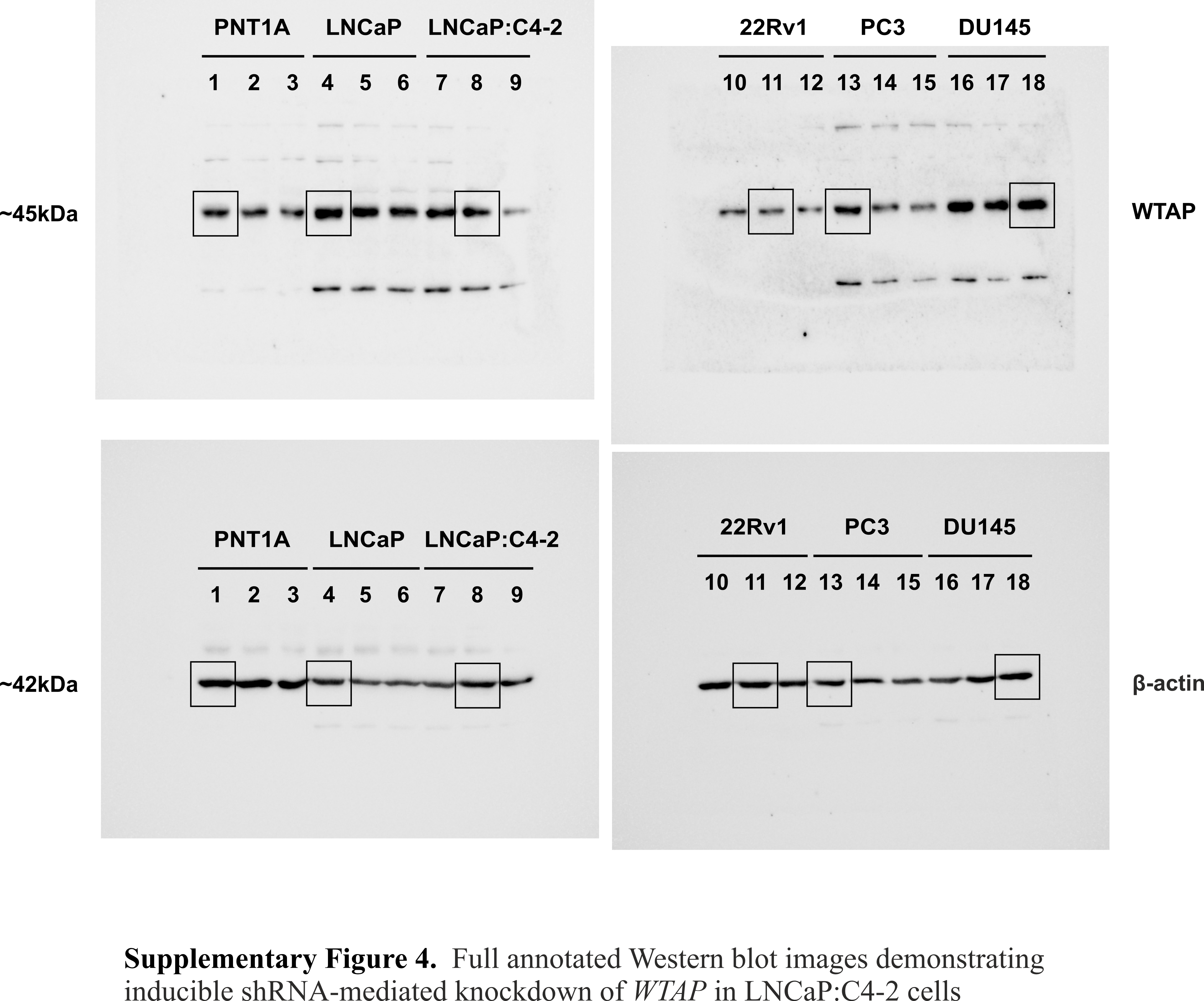

Supplement: Supplementary file 1 [file DataSheet1.zip › Supplementary figures/Supplementary_figure_4.tif]

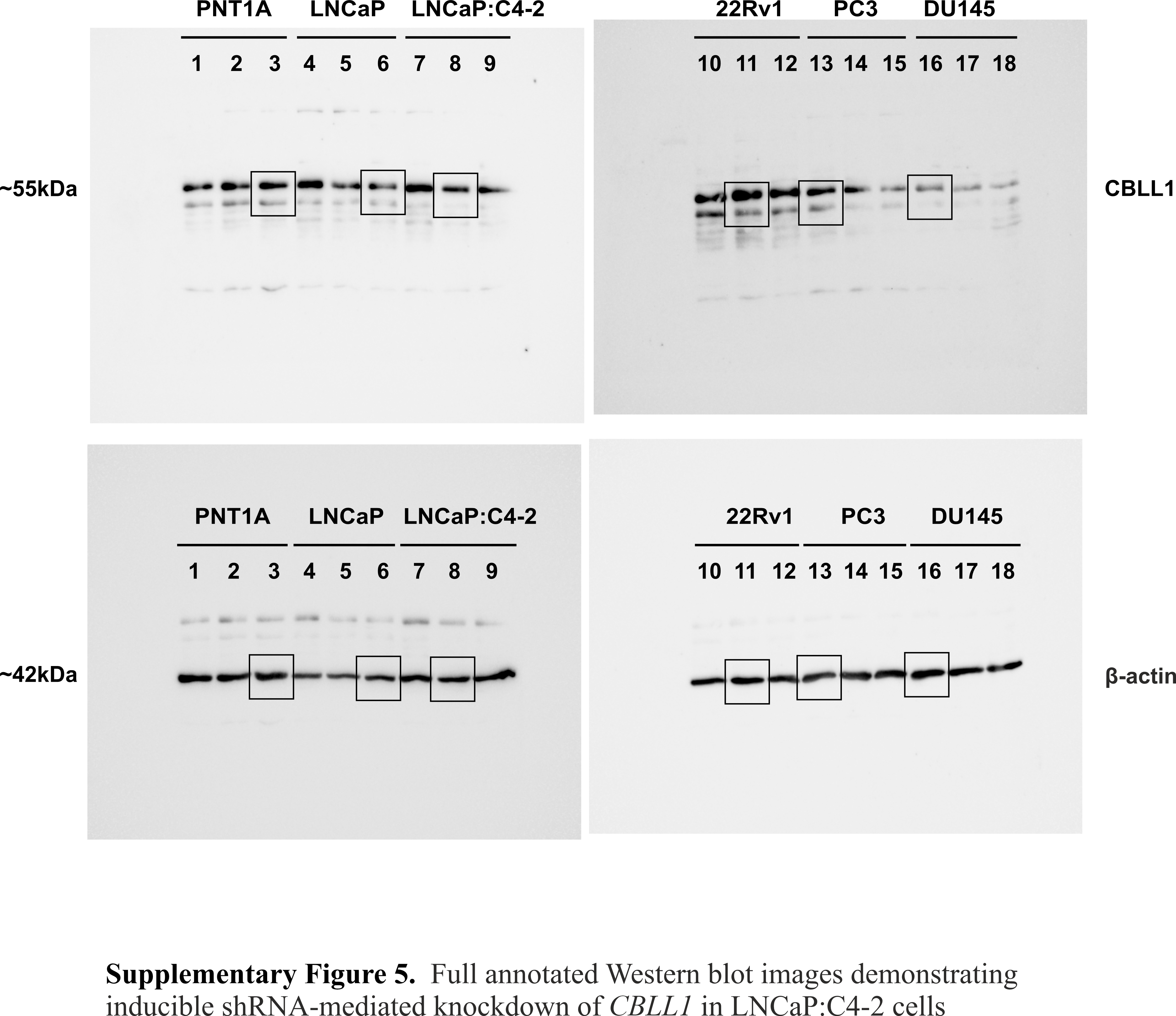

Supplement: Supplementary file 1 [file DataSheet1.zip › Supplementary figures/Supplementary_figure_5.tif]

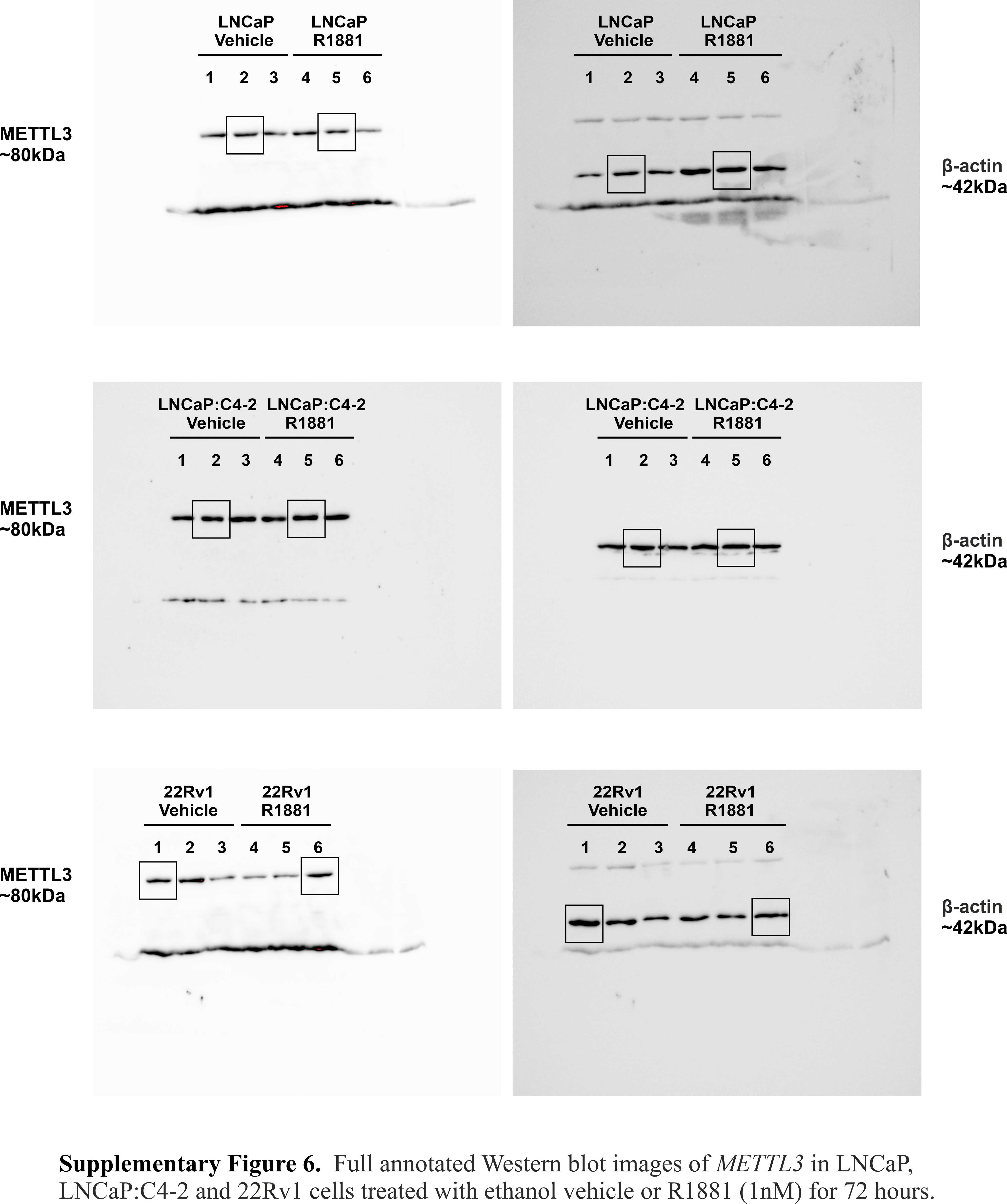

Supplement: Supplementary file 1 [file DataSheet1.zip › Supplementary figures/Supplementary_figure_6.tif]

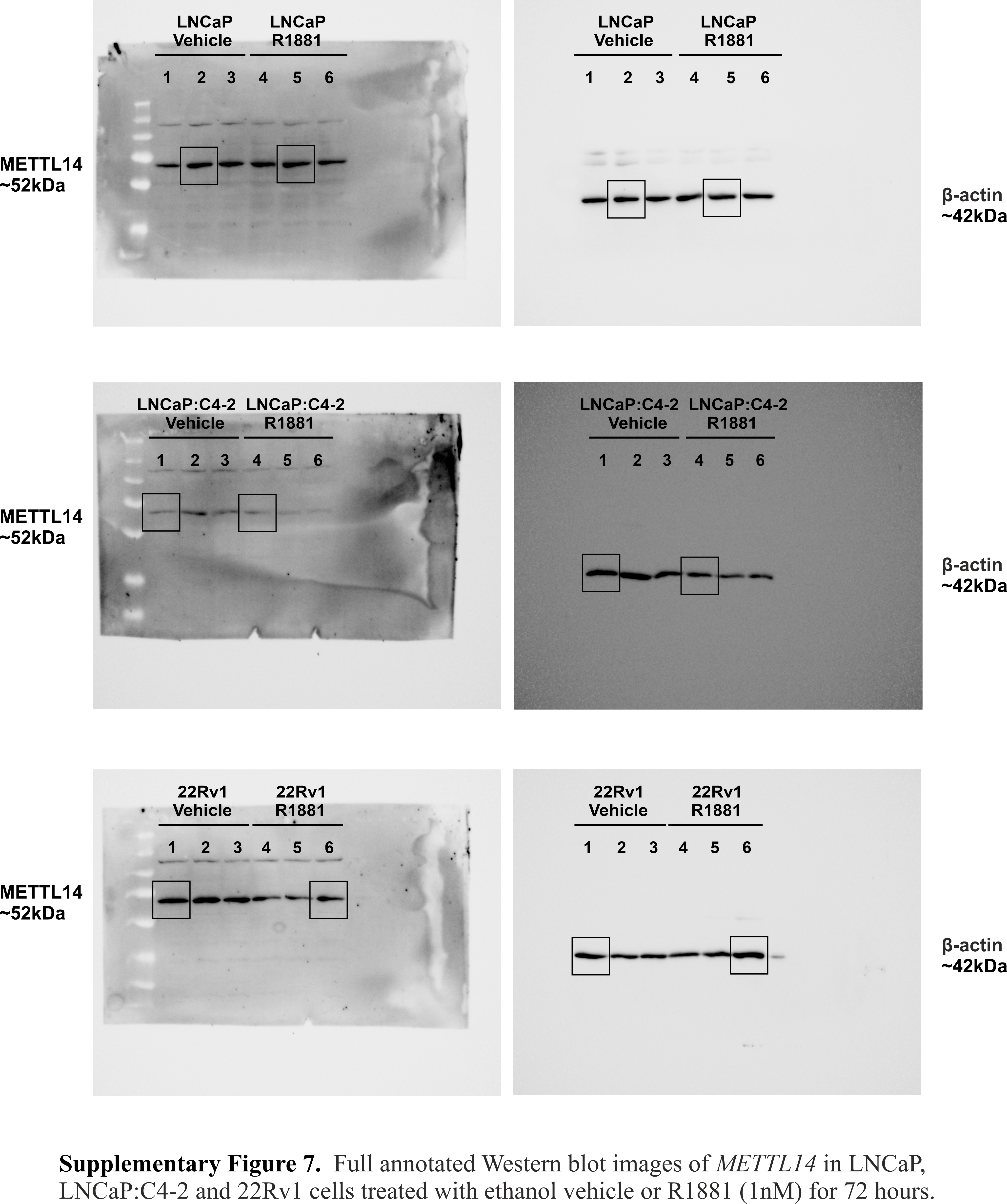

Supplement: Supplementary file 1 [file DataSheet1.zip › Supplementary figures/Supplementary_figure_7.tif]

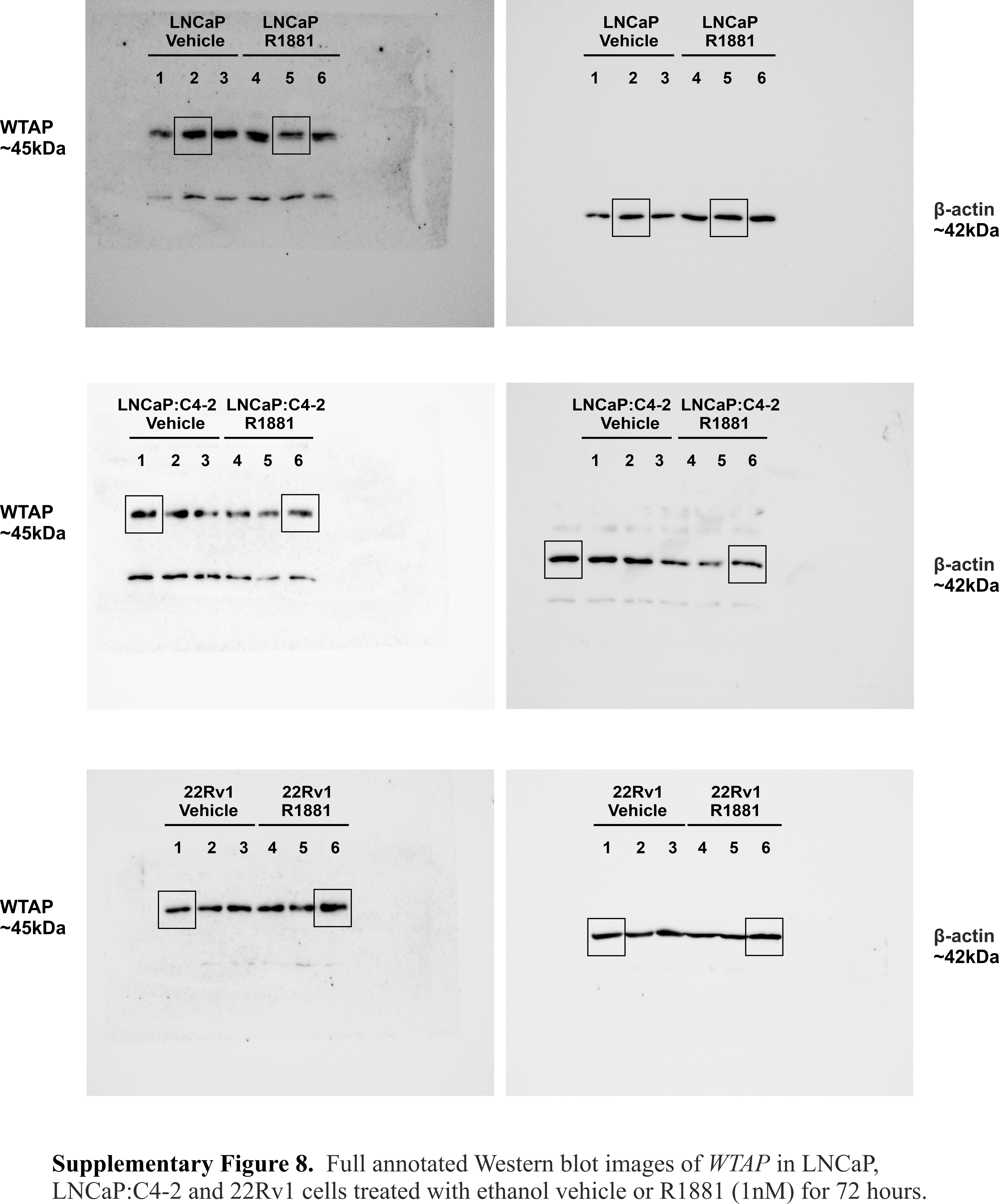

Supplement: Supplementary file 1 [file DataSheet1.zip › Supplementary figures/Supplementary_figure_8.tif]

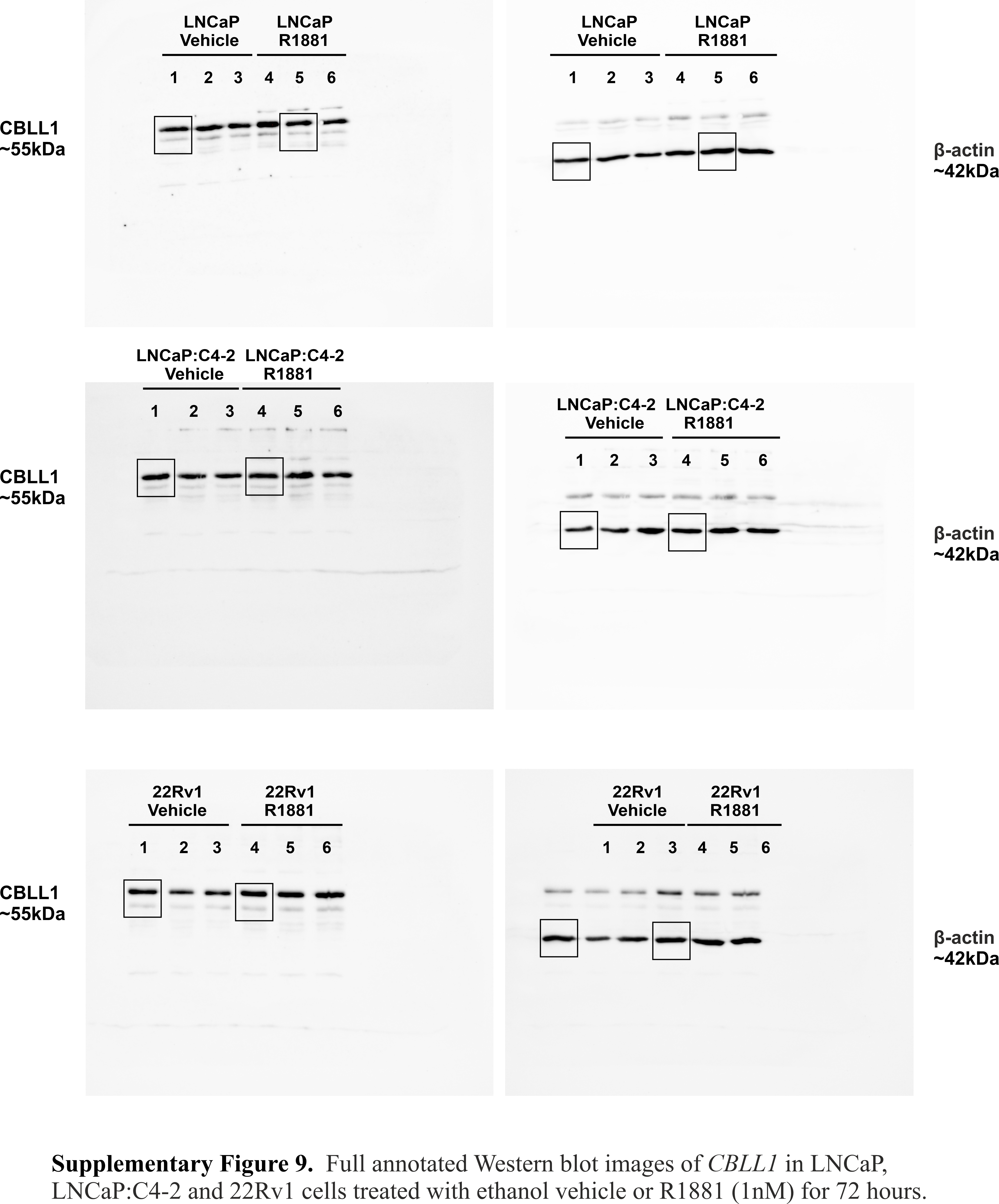

Supplement: Supplementary file 1 [file DataSheet1.zip › Supplementary figures/Supplementary_figure_9.tif]
